# Diastereoselective Synthesis of 2′-Dihalopyrimidine Ribonucleoside Inhibitors of Hepatitis C Virus Replication

**DOI:** 10.1021/acsomega.1c06174

**Published:** 2021-12-22

**Authors:** Longhu Zhou, Hongwang Zhang, Chengwei Li, Coralie De Schutter, Ozkan Sari, Seema Mengshetti, Shaoman Zhou, Mahesh Kasthuri, Steven J. Coats, Raymond F. Schinazi, Franck Amblard

**Affiliations:** Center for AIDS Research, Laboratory of Biochemical Pharmacology, Department of Pediatrics, Emory University School of Medicine, and Children’s Healthcare of Atlanta, 1760 Haygood Drive, Atlanta, Georgia 30322, United States

## Abstract

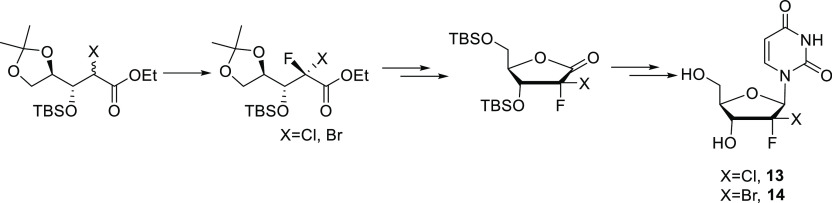

We present a newly developed synthetic route to 2-bromo-2-fluoro ribolactone based on our published 2-chloro-2-fluoro ribolactone synthesis. Stereoselective fluorination is key to controlling the 2-diastereoselectivity. We also report a substantially improved glycosylation reaction with both the 2-bromo-2-fluoro and 2-chloro-2-fluoro sugars. These improvements allowed us to prepare 2′-dihalo nucleosides **13** and **14** in an overall 15–20% yield.

## Introduction

**Figure 1:**
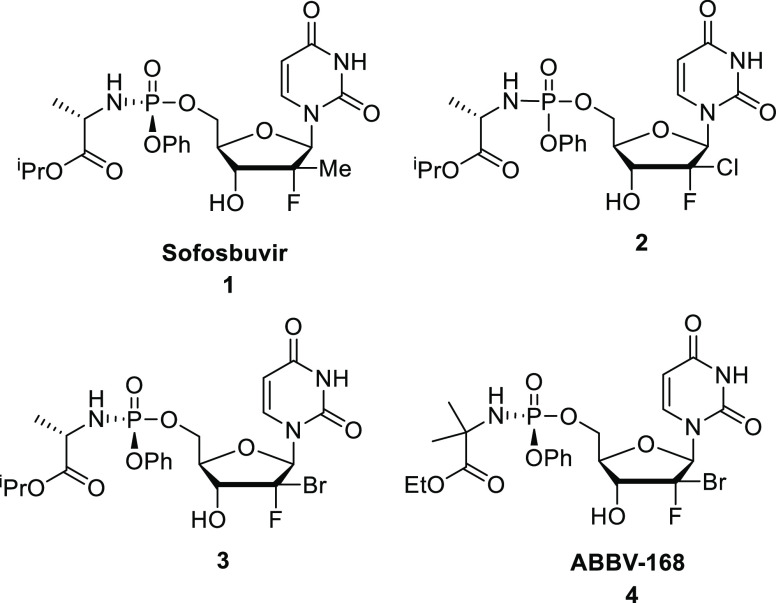
Structure of sofosbuvir and 1- and 2′-dihalo nucleoside analogues **2**, **3**, and **4**.

Hepatitis C virus (HCV) infection is a global problem with World Health Organization’s (WHO’s) estimate of worldwide chronic HCV infections of at least 71 million people. It is estimated that in 2015 there were 1.75 million newly infected patients, 70% of whom will develop chronic hepatitis. HCV is a major cause of all liver cancer cases and also the leading cause of liver transplants in the developed world.^1^ Approximately 400,000 people died in 2016 from hepatitis C, mostly from cirrhosis (326,000) and hepatocellular carcinoma.^2^ Several antiviral drugs have been developed for the treatment of HCV since the discovery of the virus in 1989. Until 2011, the combination of pegylated interferon-α (PEGIFN) with ribavirin was the treatment of choice,^3,4^ but it had limited efficacy, significant side effects, and cure rates around 50% for genotype 1 patients. Since then, several direct-acting antivirals (DAAs) targeting the viral NS3/4A protease and NS5A protein were discovered, which exhibited high efficiency for treating genotype 1 or 4 HCV-infected persons but generally showed a low barrier to the selection of the resistance virus.^5^ Significant improvement was achieved with the discovery of sofosbuvir (**1**), a nucleoside prodrug inhibitor of HCV RNA-dependent RNA polymerase (RdRp), which in combination with the NS3/4A protease inhibitor voxilaprevir and the NS5A inhibitor velpatasvir leads to the development of Vosevi, an all-oral, pan-genotypic, single-tablet regimen for chronic HCV infection.^6^ Even though a new 8 week combination regimen (glecaprevir and pibrentasvir) was approved in 2019, there is still a need to develop a novel DAA that possesses a pan-genotypic more efficacious improved safety profile and a high barrier to resistance in order to develop new ultrashort combination therapies.^7^ During the past 15 years, many attempts were made to modify natural nucleosides to identify selective and potent nucleoside analogues for the treatment of HCV.^8^ However, despite the fact that a multitude of modified nucleoside analogues reached human clinical trials, the 2′-modified uridine derivative sofosbuvir (**1**) remains the only FDA-approved nucleoside analogue for the treatment of HCV (Figure 1).

**Scheme 1:**
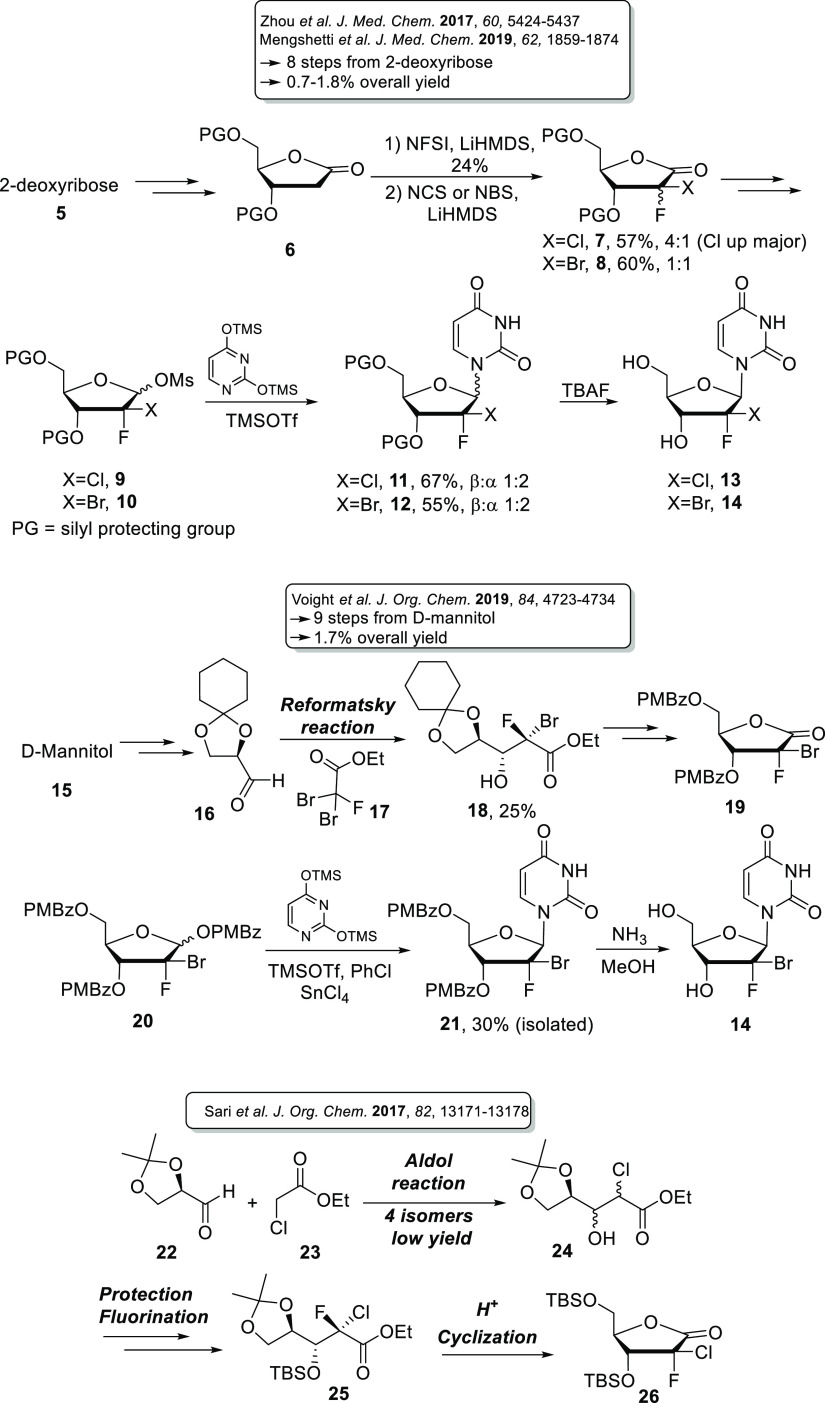
Synthetic Routes to (2*S*)-2-Halo-2-fluoro Nucleoside Derivatives **13** and **14** and Key (2*S*)-2-Chloro-2-fluoro Ribolactone **26**

We, and others, recently showed that (*S*)-2′-chloro-2′-fluoro-deoxyribouridine and (*S*)-2′-bromo-2′-fluoro-deoxyribouridine prodrugs **2**, **3**, and **4**(9,10) were non-toxic pan-genotypic anti-HCV agents (Figure 1). Because of their favorable antiviral profile, larger quantities of these compounds will be required for further *in vitro* and *in vivo* evaluations. To date, the synthesis of such (2′*S*)-Br (or Cl), 2′-F nucleosides remains challenging, mainly due to the fact that there are no good methods to introduce the two halogen atoms at the 2′-position and also because the glycosylation reaction is not stereoselective. Indeed, in our original approach, electrophilic halogenation of a 2-deoxyribolactone with *N*-fluorobenzenesulfonimide and then *N*-chloro- or *N*-bromosuccinimide in the presence of a base, led to a mixture of difficult to separate isomers in ratios ranging from 1:1 to 4:1, while the glycosylation step under various Vorbrügen-type conditions gave the undesired α-anomer as, again, a difficult to separate major coupling product (Scheme 1).

After the completion of our work, Voight et al. (Scheme 1) published an approach for the large-scale synthesis of ABBV-168 (**4**) in which the introduction of the two halogens is achieved through the Reformatsky reaction.^11^ Despite the fact that the pure isomer **18** could be easily isolated *via* crystallization, the yield of this reaction was quite low (25%). The glycosylation step was also an unresolved issue since compound **21** was only obtained in 30% yield.

With overall yields between 0.7 and 1.8%, both approaches were suboptimal, and substantial improvements were required for large-scale synthesis. Herein, we wish to report the optimization and extension of our recently published approach for the preparation of (2*S*)-2-chloro-2-fluoro ribolactone **26***via* pivotal aldol and diastereoselective fluorination reactions (Scheme 1).^12^

**Scheme 2:**
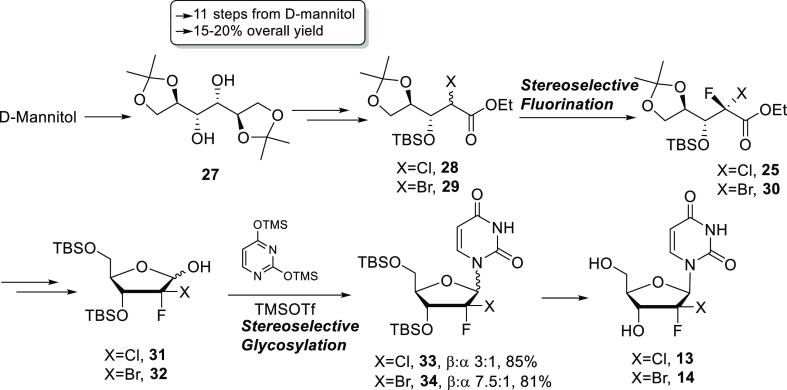
New Optimized Route to (2*S*)-2-Halo-2-fluoro Nucleoside Derivatives **13** and **14**

As summarized in Scheme 2, in the work reported herein, we developed a stereoselective fluorination that worked equally well with α-chloro^12^ and α-bromo esters. We also substantially improved the glycosylation reactions with both the 2-bromo-2-fluoro and 2-chloro-2-fluoro^12^ sugars. These improvements allowed us to prepare 2′-dihalo nucleosides **13** and **14** in an overall 15–20% yield despite adding two to three steps over our previous routes (Scheme 2).

## Results and Discussion

**Scheme 3:**
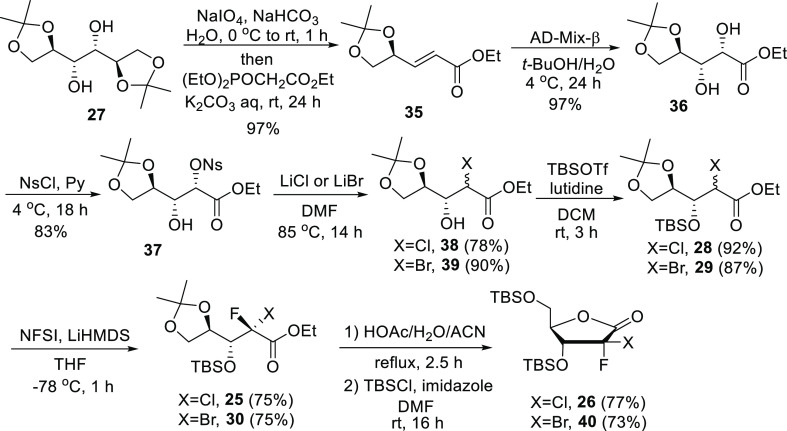
Synthesis of (2*S*)-2-Halo-2-fluorodeoxylactones **26** and **40**

The one-pot, two-step synthesis of chiral pentenoate ester **35** was accomplished *via* oxidative cleavage of commercially available 1,2:5,6-di-*O*-isopropylidene-D-mannitol **27** with sodium periodate, followed by a Knoevenagel-Doebner-type condensation reaction with commercially available triethyl α-phosphonoacetate at 0 °C in near quantitative yield (Scheme 3).^13^ Treatment of α,β-unsaturated ester **35** with AD-mix-β^14^ afforded the desired cis diol **36**, in which the more acidic α-hydroxyl group was selectively activated as a nosylate by reaction with nosyl chloride in pyridine.^15^ Displacement of the nosylate group with lithium chloride or lithium bromide in dimethylformamide or tetrahydrofuran afforded halo derivatives **38** and **39** in 78 and 90% yields, respectively.^16^ It should be noted that these displacement conditions result in some scrambling at the newly formed α-halo stereocenter.

Hydroxyl protection of **38** and **39** was first attempted with *tert*-butyldimethylsilyl chloride under a variety of conditions but always resulted in incomplete protection. Ultimately, it was discovered that the reaction with *tert*-butyldimethylsilyl trifluoromethanesulfonate in the presence of 2,6-lutidine gave a complete conversion, cleanly providing compounds **28** and **29**. Subsequent diastereoselective introduction of fluorine was accomplished using lithium bis(trimethylsilyl)amide and *N*-fluorobenzenesulfonimide as a source of fluorine. It is worth noting that **25** and **30** were obtained as single isomers and that unlike what was observed with previous methods,^12,17^ no β-elimination of *tert*-butyldimethylsiloxide occurred during the fluorination reaction. Finally, the key dihaloribolactones **26** and **40** were readily obtained by cyclization in the presence of acetic acid in water/acetonitrile at reflux, followed by 5-*tert*-butyldimethylsilyl protection with *tert*-butyldimethylsilyl chloride in dimethylformamide.^18^ With overall yields between 32 and 34%, these new procedures to synthesize the key protected dihalo lactones **26** and **40** are 3 to 5 times more efficient versus previously published approaches, respectively.

**Scheme 4:**
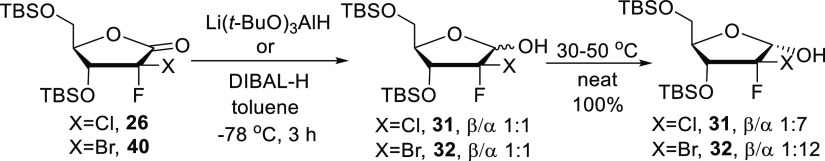
Reduction and Dynamic Crystallization of α-Lactol **31** and **32**

We then turned our attention to the glycosylation step, which is one of the major limitations in the existing synthetic routes. Indeed, Vorbrüggen-type condensation (S_N_1 mechanism) resulted in poor diastereoselectivities with β/α anomer ratios between 1/2^10^ and 1/1.^11^ We, therefore, sought to overcome this selectivity issue by introducing the nucleobase *via* an S_N_2-type reaction. The literature shows that addition of a silylated uracil (without a Lewis acid) on a pure α-1-chloro or -bromo sugar can lead selectively to the formation of β nucleoside anomers.^19,20^ Interestingly, in 2011, Reddy et al.^19^ were able to isomerize a 2/1 (β/α) 1-lactol mixture to the thermodynamically more stable β-lactol (ratio 20/1) by simply heating the neat material at 50 °C for 20 h; proceeding presumably by crystallization induced dynamic resolution.^21^ From that enriched β lactol, the formation of a 1-α-chloro sugar intermediate (7/1 ratio) was achieved through a modified Appel reaction (triphenylphosphine/*N*-chlorosuccinimide) before introduction of the nucleobase *via* an S_N_2-type reaction. Based on this precedent, we took lactol **32** (∼1/1, β/α), obtained after reduction of lactone **40** with lithium tri-*tert*-butoxyaluminum hydride, and heated the neat material at 45 °C for 7 days (Scheme 4). By doing so, the anomer ratio went up to 12:1, as determined by proton NMR integration. However, after careful characterization, we realized that, unlike what was observed by Reddy et al. in the case of a 2-Me-2-F sugar (β-isomer predominant after isomerization), the α-lactol was the major isomer in our case. The anomers were readily assigned 1-position stereochemistry through both proton and fluorine NMR where the 1-proton or 2-fluorine coupling constants were always larger when the coupling partner was in the anti-configuration. The α-configuration of the lactol was further confirmed when we observed the selective formation of the α-nucleoside after bromination of the lactol *via* the Appel reaction (triphenylphosphine/carbon tetrabromide) and further bromine displacement with silylated uracil. Finally, it is worth noting that while stable when kept as a solid, the α-lactol isomer quickly anomerizes once in solution (as determined by NMR analysis in CDCl_3_), which necessitated lower temperatures and quick reaction times when working with these 1-lactols. Reddy et al. had also observed a solvent-dependent rate of anomerization with a preference for β; in contrast, at 1 h in CDCl_3_, acetone-*d*_6_, and methanol-*d*_4_, we found β/α ratios less than 1/2, but always favoring the α-anomer. Like Reddy et al., we also found more anomer stability in DMSO-*d*_6_, but in our case, the β/α ratio at 1 day was 1/6. With lactol **32** as a 1/12 β/α mixture in hand, we then focused on the key glycosylation step. Lactol **32** was initially activated with a mesyl group, but unfortunately, the resulting 1-mesylate was too stable and could not be displaced when treated with a solution of silylated uracil (S_N_2 conditions). We hypothesized that a better leaving group would be required in order for the glycosylation to be performed and decided to introduce a triflate group instead. Thus, to a solution of **32** (1/12 β/α mixture) in dichloromethane was added sequentially at −78 °C triethylamine (Et_3_N) and then trifluoromethanesulfonic anhydride (Tf_2_O). The triflate intermediate **41** was found to be too reactive to be isolated, so after stirring for 1 h at −78 °C, a precooled solution of silylated uracil was added. After 1 h at −78 °C, followed by 4 h at room temperature (rt), we isolated nucleoside **34** in 88% yield but as a 1/1 mixture of anomers (Table 1, entry 1).

**Table 1:**
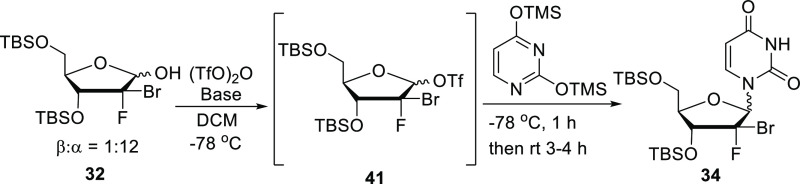
Glycosylation Optimization

entry	base	34 (β/α)^a^	isolated yield (%)
1	Et_3_N^b^	1:1	85
2	Et_3_N^c^	1.7:1	85
3	Et_3_N^d^	3.5:1	88
4	NMI	6:1	85
5	DBU	6:1	75
6	DIPEA	7.5:1	88

a Ratio determined by ^1^H NMR.

b Addition of Et_3_N at −78 °C, followed by the addition of (TfO)_2_O.

c Addition of (TfO)_2_O at −78 °C, followed by the addition of Et_3_N after 20 min.

d Simultaneous addition of (TfO)_2_O and Et_3_N at −78 °C.

**Scheme 5:**
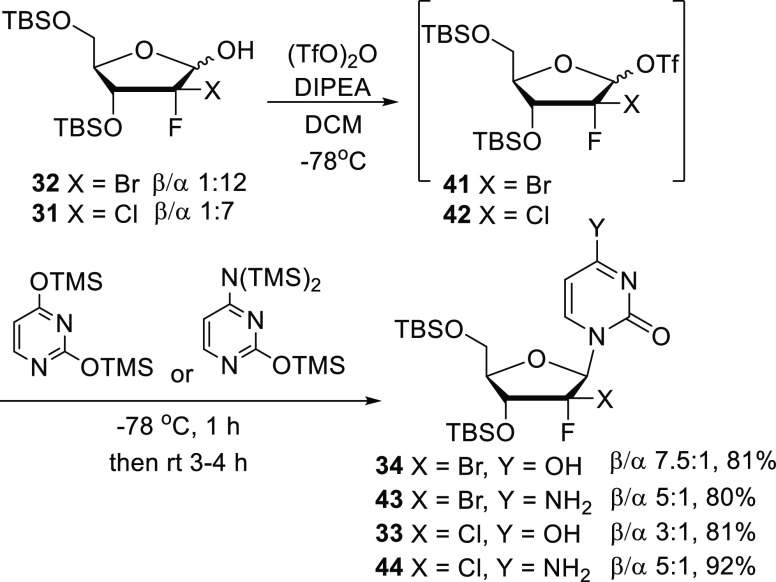
Extension of Our Glycosylation Approach to the Synthesis of Nucleoside Analogues **34**, **43**, **33**, and **44**

Interestingly, this β/α ratio was improved to 1.7/1 when Tf_2_O was added first and the solution stirred for 20 min before addition of Et_3_N (Table 1, entry 2). Ultimately, we found that the ratio could further be improved when both Tf_2_O and Et_3_N were added at the same time (Table 1, entry 3). Under these conditions, compound **34** was isolated as a 3.5/1 mixture, in 88% yield. Based on these preliminary results, the use of different organic bases was then evaluated to further optimize the glycosylation reaction. Replacement of Et_3_N with *N*-methylimidazole (Table 1, entry 4) or 1,8-diazabicyclo[5.4.0]undec-7-ene (Table 1, entry 5) leads to the formation of compound **34** in a 6/1 ratio, while the use of *N*,*N*-diisopropylethylamine (Table 1, entry 6) gave us the best outcome with compound **34** being isolated as a 7.5/1 mixture (88% yield). It is worth noting that the glycosylation was achieved under these final conditions on a 5 g scale without any effect on the yield or anomer ratio. Replacing silylated uracil with silylated cytosine, cytosine nucleoside **43** could also be obtained predominantly as a beta isomer (5/1 ratio) (Scheme 5). In addition, treatment of 2-Cl-2-F lactol **31** (7/1) under the same conditions allowed us to isolate in excellent yields the corresponding uracil and cytosine derivatives **33** and **44** as 3/1 and 5/1 mixtures, respectively (Scheme 5). Compounds **33**, **34**, **43**, and **44** were deprotected using the method we previously described^9b,10^ (either with Et_3_N·3HF or *tetra*-*N*-butylammonium fluoride) to afford the corresponding β nucleoside analogues (>95% purity).

## Conclusions

In summary, we present herein an improved route to 2-dihalo ribolactones **13** and **14**, which hinged on a completely diastereoselective fluorination reaction. These ribolactones were then utilized to prepare all four pyrimidine nucleosides in a newly optimized glycosylation reaction that took advantage of a crystallization-induced dynamic resolution of the intermediate 1-lactols **41** and **42**. The improved yields and diastereoselectivity in both phases of the synthesis allowed us to prepare the final nucleosides more efficiently and with elimination of difficult diastereomeric mixture separations.

## Experimental Section

Anhydrous solvents were purchased from Millipore Sigma (Milwaukee, WI). All commercially available reagents were used without further purification. Reagents were purchased from commercial sources. All the reactions were carried out under nitrogen in oven-dried glassware unless otherwise noted. Thin-layer chromatography was performed on Analtech GHLF silica gel plates. Column chromatography was accomplished on a Combiflash Rf200 or via reverse-phase high-performance liquid chromatography. ^1^H, ^13^C, and ^19^F NMR spectra were recorded on a Bruker Ascend 400 spectrometer at rt (400, 101, and 377 MHz), and residual proton solvent signals were used as internal standards. Deuterium exchange and decoupling experiments were utilized to confirm proton assignments. NMR processing was performed with MestReNova (Mestrelab Research, Compostela, Spain) version 14.1.1 24571 or Topspin (Bruker, Berlin, Germany) version 3.5. Signal multiplicities are represented by s (singlet), d (doublet), dd (doublet of doublets), t (triplet), q (quadruplet), br (broad), bs (broad singlet), and m (multiplet). Coupling constants (*J*) are in hertz (Hz). Mass spectra were determined on a Waters Acquity UPLC LC spectrometer using an SQ detector with electrospray ionization (ESI). The purity of final compounds was determined to be >95% using ultraperformance liquid chromatography (UPLC) analyses performed on a Waters Acquity UPLC System with a Kinetex LC column (2.1 mm Å, 50 mm, 1.7 μm, C18, 100 Å) and further supported by clean NMR spectra. The mobile phase flow was 0.4 mL/min with a 1.20 min gradient from 95% aqueous media (0.05% formic acid) to 95% CH_3_CN (0.05% formic acid) and a 4.5 min total acquisition time. Photodiode array detection was from 190 to 360 nm.

### Ethyl (*S*,*E*)-3-(2,2-Dimethyl-1,3-dioxolan-4-yl) Acrylate (**35**)

To a slurry of 18.9 g (72 mmol) of 1,2:5,6-di-*O*-isopropylidene-*D*-mannitol (**27**) in 150 mL of 5% aqueous NaHCO_3_ at 0 °C was added dropwise a solution of 18.9 g (88.5 mmol) of NaIO_4_ in 150 mL of water. The bath was removed, and the mixture was stirred for 1 h. The mixture was cooled to 0 °C, followed by the addition of triethyl α-phosphonoacetate (67.8 g, 300 mmol) and 450 mL of 6 M K_2_CO_3_. The reaction mixture was allowed to warm to rt and was stirred for 24 h. The reaction mixture was extracted four times with CH_2_Cl_2_, and the combined extracts were dried over Na_2_SO_4_, filtered, and concentrated under reduced pressure. The residue was purified by chromatography with a gradient of hexane to 50% ether-hexane to afford 28.0 g (97%) of *E*-isomer (**35**) and 0.7 g (2.4%) of *Z*-isomer. ^1^H NMR (400 MHz, CDCl_3_): δ 6.86 (dd, *J* = 15.6, 5.6 Hz, 1H), 6.08 (dd, *J* = 15.6, 0.8 Hz, 1H), 4.64 (m, 1H), 4.17 (q, *J* = 7.2 Hz, 2H), 4.15 (m, 1H), 3.66 (dd, *J* = 8.2, 7.2 Hz, 1H), 1.43 (s, 3H), 1.39 (s, 3H), 1.27 (t, *J* = 7.2 Hz, 3H). ^13^C NMR (101 MHz, CDCl_3_): δ 165.92, 144.56, 122.36, 110.09, 74.87, 68.73, 60.49, 26.38, 25.66, 14.13; high-resolution mass spectrometry (HRMS) (ESI): *m*/*z* [M + H]^+^ calcd for C_10_H_17_O_4_, 201.1127; found, 201.1118.

### Ethyl (2*R*,3*S*)-3-((*R*)-2,2-Dimethyl-1,3-dioxolan-4-yl)-2,3-dihydroxypropanoate (**36**)

To a solution of ^t^BuOH (150 mL) and H_2_O (150 mL) was added AD-mix-β (42.0 g) at rt. When the two-phase solution became clear (the bottom phase is pale yellow), methanesulfonamide (2.85 g, 30.0 mmol) was added. The mixture was cooled to 0 °C, and when the solution started to form a suspension, **35** (6.0 g, 30.0 mmol) was added. The reaction mixture was stirred at 4 °C for 24 h until completion. Na_2_S_2_O_3_ (45 g) was added at 4 °C, and the mixture was stirred from 4 °C toward rt for 60 min. Ethyl acetate (200 mL) was added and separated. The water phase was extracted with ethyl acetate (60 mL × 3). The extracts were dried over Na_2_SO_4_ and concentrated *in vacuo*. The residue was purified by silica gel column chromatography (0 to 50% EtOAc in hexane) to give **36** (6.8 g, 96.8%) as a colorless oil. ^1^H NMR (400 MHz, CDCl_3_): δ 4.43 (d, *J* = 4.6 Hz, 1H), 4.32 (q, *J* = 7.2 Hz, 2H), 4.15–4.04 (m, 3H), 3.88 (m, 1H), 3.14 (m, 1H), 2.27–2.23 (m, 1H), 1.45 (s, 3H), 1.37 (s, 3H), 1.33 (t, *J* = 7.2 Hz, 3H); ^13^C NMR (101 MHz, CDCl_3_): δ 173.42, 109.38, 75.20, 72.97, 70.40, 66.77, 62.13, 26.82, 25.13, 14.05; HRMS (ESI): *m*/*z* [M + H]^+^ calcd for C_10_H_19_O_6_, 235.1182; found, 235.1191.

### Ethyl (2*R*,3*R*)-3-((*R*)-2,2-Dimethyl-1,3-dioxolan-4-yl)-3-hydroxy-2-(((4-nitrophenyl)sulfonyl)oxy) Propanoate (**37**)

To a stirred solution of **36** (6.8 g, 29.06 mmol) in pyridine (125 mL) at 0 °C was added NaCl (7.4 g, 33 mmol). After stirring at 4 °C for 18 h, the mixture was quenched with water (5 mL) at 4 °C, treated with Et_2_O (500 mL), and washed with pre-cooled 1 M aq. KHSO_4_ (100 mL × 3) and saturated brine (100 mL). The organic layer was dried over Na_2_SO_4_ and concentrated *in vacuo*. The residue was purified by silica gel column chromatography (0 to 60% EtOAc in hexane) to give **37** (9.9 g, 83%) as a pale-yellow oil. ^1^H NMR (400 MHz, CDCl_3_): δ 8.38 (d, *J* = 7.0 Hz, 2H), 8.20 (d, *J* = 7.0 Hz, 2H), 5.27 (d, *J* = 2.0 Hz, 1H), 4.25 (q, *J* = 7.1 Hz, 2H), 4.07–3.98 (m, 3H), 3.85–3.81 (m, 1H), 2.54 (br s, 1H), 1.37 (s, 3H), 1.28 (t, *J* = 7.1 Hz, 3H), 1.20 (s, 3H); ^13^C NMR (101 MHz, CDCl_3_): δ 166.98, 150.83, 141.94, 129.60, 124.16, 109.86, 78.63, 74.09, 72.76, 66.63, 62.64, 26.82, 24.88, 13.98; HRMS (ESI): *m*/*z* [M + H]^+^ calcd for C_16_H_22_NO_10_S, 420.0964; found, 420.0958.

### Ethyl (3*R*)-2-Chloro-3-((*R*)-2,2-dimethyl-1,3-dioxolan-4-yl)-3-hydroxypropanoate (**38**)

To a stirred solution of **37** (4.8 g, 11.4 mmol) in DMF (60 mL) was added LiCl (970 mg, 22.8 mmol). The mixture was heated to 85 °C and stirred for 14 h. After the mixture was treated with Et_2_O at 0 °C, the ethereal solution was washed with pre-cooled 1 M aq. KHSO_4_ (50 mL × 3) and saturated brine (50 mL), dried over Na_2_SO_4_, and concentrated *in vacuo*. The residue was purified by silica gel column chromatography (0 to 35% ethyl ether in hexane) to give **38** (2.25 g, 78%) as a colorless oil. ^1^H NMR (400 MHz, CDCl_3_): δ 4.73 (d, *J* = 1.8 Hz, 0.5H), 4.5 (d, *J* = 4.1 Hz, 0.5H), 4.31 (q, *J* = 7.1 Hz, 2H), 4.20–4.01 (m, 3H), 2.96 (d, *J* = 7.3 Hz, 0.5H), 2.73 (d, *J* = 6.4 Hz, 0.5H), 1.62–1.32 (m, 9H); ^13^C NMR (101 MHz, CDCl_3_): δ 168.77, 167.91, 110.26, 109.85, 75.38, 74.85, 74.26, 72.82, 72.65, 66.91, 66.52, 66.34, 62.60, 62.43, 71.71, 59.17, 57.54, 26.87, 26.54, 25.95, 25.19, 25.01, 4.06, 13.92; HRMS (ESI): *m*/*z* [M + H]^+^ calcd for C_10_H_18_ClO_5_, 253.0843; found, 253.0839.

### Ethyl (3*R*)-3-((*tert*-Butyldimethylsilyl)oxy)-2-chloro-3-((*R*)-2,2-dimethyl-1,3-dioxolan-4-yl) Propanoate (**28**)

To a solution of **38** (3.0 g, 11.8 mmol) in DCM was added 2,6-lutidine (4.1 mL, 35.4 mmol) at 0 °C. To the mixture was added TBSOTf (4.1 mL, 17.7 mmol) dropwise at 0 °C. The resulting mixture was stirred at rt for 3 h. The reaction was quenched with pre-cooled 1 N HCl (30 mL) at 0 °C, extracted with DCM (50 mL × 3), washed with water (50 mL) and brine (50 mL), dried over Na_2_SO_4_, and concentrated in vacuum. The residue was purified by silica gel column chromatography (0–20% ethyl acetate in hexane) to give **28** (4.0 g, 92.4%) as a colorless oil. ^1^H NMR (400 MHz, CDCl_3_): δ 4.65 (d, *J* = 2.5 Hz, 0.5H), 4.59 (d, *J* = 2.64 Hz, 0.5H), 4.30–4.17 (m, 4H), 4.09–4.04 (m, 1H), 3.92–3.88 (m, 1H), 1.43 (s, 1.5H), 1.39 (s, 1.5H), 1.34–1.32 (m, 6H), 0.89 (s, 4.5H), 0.87 (s, 4.5H), 0.18 (s, 1.5H), 0.14 (s, 1.5H), 0.11 (s, 1.5H), 0.04 (s, 1.5H); ^13^C NMR (101 MHz, CDCl_3_): δ 168.35, 166.83, 109.41, 109.28, 75.95, 75.79, 75.54, 73.99, 66.63, 66.57, 62.39, 62.21, 61.25, 60.41, 26.69, 26.53, 25.68, 25.65, 25.07, 25.03, 18.16, 18.03, 14.06, 13.97, −4.35, −4.38, −4.45, −4.69. HRMS (ESI): *m*/*z* [M + H]^+^ calcd for C_16_H_32_ClO_5_Si, 367.1708; found, 367.1697.

### Ethyl (2*S*,3*R*)-3-((*tert*-Butyldimethylsilyl)oxy)-2-chloro-3-((*R*)-2,2-dimethyl-1,3-dioxolan-4-yl)-2-fluoropropanoate (**25**)

To a solution of **28** (3.95 g, 10.7 mmol) and NSFI (5.08 g, 16.1 mmol) in THF (50 mL) was added LiHMDS (16.1 mL, 16.1 mmol) dropwise at −78 °C. The mixture was stirred at −78 °C for 1 h, and LDA solution (3.5 mL, 3.5 mmol) was added dropwise at this temperature. The reaction mixture was then stirred from −78 to −10 °C (∼1 h). The reaction mixture was quenched with saturated NH_4_Cl (50 mL) and ethyl acetate (200 mL) at −78 °C. The organic layer was washed with saturated NH_4_Cl (50 mL × 2), water (50 mL), and brine (50 mL); dried over Na_2_SO_4_; and concentrated *in vacuo*. The residue was purified by silica gel column chromatography (0–20% ethyl acetate in hexane) to give **25** (3.1 g, 75%) as a colorless oil.

^1^H NMR (400 MHz, CDCl_3_): δ 4.37–4.26 (m, 4H), 4.04 (dd, *J* = 8.2, 6.6 Hz, 1H), 3.92 (dd, *J* = 8.1, 6.4 Hz, 1H), 1.41 (s, 3H), 1.36 (t, *J* = 7.1 Hz, 3H), 1.32 (s, 3H), 0.92 (s, 9H), 0.19 (s, 3H), 0.16 (s, 3H); ^19^F NMR (377 MHz, CDCl_3_): δ −127.14 (d, *J* = 18.1 Hz). HRMS (ESI): *m*/*z* [M + H]^+^ calcd for C_16_H_31_ClFO_5_S, 385.1613; found, 385.1619.

### (3*S*,4*R*,5*R*)-4-((*tert*-Butyldimethylsilyl)oxy)-5-(((*tert*-butyldimethylsilyl)oxy)methyl)-3-chloro-3-fluorodihydrofuran-2(3*H*)-one (**26**)

A solution of **25** (2.9 g, 7.5 mmol) in HOAc/H_2_O/ACN (4:1:10, 15 mL) was refluxed with a 100 °C oil bath for 2.5 h (monitored by TLC for completion). The solvent was removed under reduced pressure and co-evaporated with toluene (10 mL × 3). The residue was dissolved in DMF (20 mL) and cooled to 0 °C, and imidazole (1.22 g, 18 mmol) was added, followed by TBSCl (2.26 g, 15 mmol). The reaction mixture was stirred toward rt overnight to completion. The reaction mixture was quenched with water and extracted with ethyl ether (200 mL). The organic layer was washed with water (30 mL × 3), saturated NH_4_Cl (50 mL), and brine (50 mL); dried over Na_2_SO_4_; and concentrated in vacuum. The residue was purified by silica gel column chromatography (0–8% ethyl acetate in hexane) to give **26** (2.4 g, 77%) as a colorless oil.

^1^H NMR (400 MHz, CDCl_3_): δ 4.59 (dd, *J* = 11.9, 5.8 Hz, 1H), 4.37–4.32 (m, 1H), 3.98 (dd, *J* = 12.1, 3.9 Hz, 1H), 3.85 (dd, *J* = 12.1, 3.1 Hz, 1H), 0.93 (s, 9H), 0.90 (s, 9H), 0.22 (s, 3H), 0.17 (s, 3H), 0.10 (s, 3H), 0.09 (s, 3H); ^19^F NMR (377 MHz, CDCl_3_): δ −134.66 (d, *J* = 12.0 Hz); ^13^C NMR (101 MHz, CDCl_3_): δ 165.11 (d, *J* = 26.26 Hz), 102.06 (d, *J* = 260.6 Hz), 83.60, 74.06 (d, *J* = 16.2 Hz), 59.52, 25.70, 25.50, 18.20, 18.01, −4.45, −5.21, −5.48, −5.52. HRMS (ESI): *m*/*z* [M + H]^+^ calcd for C_17_H_35_ClFO_4_Si_2_, 413.1746; found, 413.1736.

### Ethyl (3*R*)-2-Bromo-3-((*R*)-2,2-dimethyl-1,3-dioxolan-4-yl)-3-hydroxypropanoate (**39**)

To a stirred solution of **37** (5.98 g, 14.3 mmol) in THF (70 mL) was added LiBr (6.4 g, 73.5 mmol). The mixture was heated to 75 °C and stirred overnight. After the mixture was treated with Et_2_O (300 mL) at 0 °C, the ethereal solution was washed with pre-cooled 1 M aq. KHSO_4_ (50 mL × 3) and saturated brine (50 mL), dried over Na_2_SO_4_, and concentrated *in vacuo*. The residue was purified by silica gel column chromatography (0–35% ethyl ether in hexane) to give **39** (3.8 g, 89.6%) as a colorless oil.

^1^H NMR (400 MHz, CDCl_3_): δ 4.66 (d, *J* = 2.2 Hz, 0.5H), 4.5 (d, *J* = 4.4 Hz, 0.5H), 4.30–4.23 (m, 2H), 4.16–4.01 (m, 3H), 3.93–3.71 (m, 1H), 3.37 (d, *J* = 8.1 Hz, 0.5H), 3.22 (d, *J* = 4.0 Hz, 0.5H), 1.41 (s, 1.5H), 1.40 (s, 1.5H), 1.34 (s, 3H), 1.32 (t, *J* = 7.1 Hz, 1.5H), 1.31 (t, *J* = 7.1 Hz, 1.5H); ^13^C NMR (101 MHz, CDCl_3_): δ 169.76, 169.07, 109.98, 109.83, 76.10, 75.49, 74.28, 71.74, 66.96, 66.52, 62.54, 62.44, 49.02, 44.56, 26.89, 26.65, 25.03, 13.84; HRMS (ESI): *m*/*z* [M + H]^+^ calcd for C_10_H_18_BrO_5_, 297.0338; found, 297.0343.

### Ethyl (3*R*)-3-((*tert*-Butyldimethylsilyl)oxy)-2-bromo-3-((*R*)-2,2-dimethyl-1,3-dioxolan-4-yl) Propanoate (**29**)

To a solution of **39** (3.8 g, 12.8 mmol) in DCM (60 mL) was added 2,6-lutidine (4.5 mL, 38.8 mmol) at 0 °C. The mixture was added TBSOTf (4.5 mL, 19.4 mmol) dropwise at 0 °C. The resulting mixture was stirred at rt for 3 h. The reaction mixture was quenched with pre-cooled 1 N HCl (30 mL) at 0 °C, extracted with DCM (50 mL × 3), washed with water (50 mL) and brine (50 mL), dried over Na_2_SO_4_, and concentrated *in vacuo*. The residue was purified by silica gel column chromatography (0–20% ethyl acetate in hexane) to give **29** (4.58 g, 87.1%) as a colorless oil.

^1^H NMR (400 MHz, CDCl_3_): δ 4.65 (d, *J* = 2.8 Hz, 0.5H), 4.90 (d, *J* = 3.8 Hz, 0.5H), 4.34–4.11 (m, 4H), 4.08–4.01 (m, 1H), 3.91–3.87 (m, 1H), 1.41 (s, 1.5H), 1.39 (s, 1.5H), 1.33–1.30 (m, 6H), 0.89 (s, 4.5H), 0.87 (s, 4.5H), 0.16 (s, 1.5H), 0.13 (s, 1.5H), 0.11 (s, 1.5H), 0.05 (s, 1.5H); ^13^C NMR (101 MHz, CDCl_3_): δ 168.11, 166.66, 109.24, 109.17, 76.59, 76.32, 74.56, 73.38, 66.49, 65.91, 62.37, 62.29, 51.19, 51.02, 26.62, 26.44, 25.70, 25.67, 25.01, 24.95, 18.17, 18.03, 13.93, −4.40, −4.43, −4.48; HRMS (ESI): *m*/*z* [M + H]^+^ calcd for C_16_H_32_BrO_5_Si, 411.1202; found, 411.1211.

### Ethyl (2*S*,3*R*)-3-((*tert*-Butyldimethylsilyl)oxy)-2-bromo-3-((*R*)-2,2-dimethyl-1,3-dioxolan-4-yl)-2-fluoropropanoate (**30**)

To a solution of **29** (4.45 g, 10.87 mmol) and NSFI (5.12 g, 16.23 mmol) in THF (60 mL) was added LiHMDS (18.5 mL, 18.5 mmol) dropwise at −78 °C. The mixture was stirred at −78 °C for 1 h, and LDA solution (2 mL, 2 mmol) was added dropwise at this temperature. The reaction mixture was then stirred from −78 to −10 °C for completion (1 h). The reaction mixture was quenched with saturated NH_4_Cl (50 mL) and ethyl acetate (200 mL) at −78 °C. The organic layer was washed with saturated NH_4_Cl (50 mL × 2), water (50 mL), and brine (50 mL); dried over Na_2_SO_4_; and concentrated in vacuum. The residue was purified by silica gel column chromatography (0–20% ethyl acetate in hexane) to give **30** (3.5 g, 75.4%) as a colorless oil.

^1^H NMR (400 MHz, CDCl_3_): δ 4.45–4.25 (m, 4H), 4.03 (dd, *J* = 8.2, 6.6 Hz, 1H), 3.92 (dd, *J* = 8.1, 6.5 Hz, 1H), 1.41 (s, 3H), 1.36 (t, *J* = 7.1 Hz, 3H), 1.32 (s, 3H), 0.93 (s, 9H), 0.22 (s, 3H), 0.17 (s, 3H); ^19^F NMR (377 MHz, CDCl_3_): δ −126.16 (d, *J* = 18.0 Hz); ^13^C NMR (101 MHz, CDCl_3_): δ 165.24 (d, *J* = 26.36 Hz), 108.77, 98.75 (d, *J* = 271.84 Hz), 77.76 (d, *J* = 20.42 Hz), 75.60 (d, *J* = 4.16 Hz), 65.48, 63.02, 25.89, 25.86, 24.50, 18.33, 13.78, −3.91, −4.08, −4.10; HRMS (ESI): *m*/*z* [M + Na]^+^ calcd for C_16_H_30_BrFNaO_5_Si, 451.0928; found, 451.0921.

### (3*S*,4*R*,5*R*)-4-((*tert*-Butyldimethylsilyl)oxy)-5-(((*tert*-butyldimethylsilyl)oxy)methyl)-3-bromo-3-fluorodihydrofuran-2(3*H*)-one (**40**)

A solution of **30** (1.54 g, 3.75 mmol) in HOAc/H_2_O/ACN (4:1:10, 8 mL) was heated under reflux in a 100 °C oil bath for 2.5 h (monitored by TLC for completion). The solvent was removed in reduced pressure and co-evaporated with toluene (10 mL × 3). The residue was dissolved in DMF (10 mL) and cooled to 0 °C, and imidazole (456 mg, 6.7 mmol) was added, followed by TBSCl (845 mg, 5.6 mmol). The reaction mixture was stirred toward rt overnight. The reaction mixture was quenched with water and extracted with ethyl ether (150 mL). The organic layer was washed with water (30 mL × 3), saturated NH_4_Cl (50 mL), and brine (50 mL); dried over Na_2_SO_4_; and concentrated *in vacuo*. The residue was purified by silica gel column chromatography (0–8% ethyl acetate in hexane) to give **40** (1.2 g, 73%) as a colorless oil.

^1^H NMR (400 MHz, CDCl_3_): δ 4.73 (dd, *J* = 8.6, 4.3 Hz, 1H), 4.41–4.37 (m, 1H), 3.99 (dd, *J* = 11.7, 5.3 Hz, 1H), 3.90 (dd, *J* = 11.7, 3.9 Hz, 1H), 0.93 (s, 9H), 0.90 (s, 9H), 0.22 (s, 3H), 0.18 (s, 3H), 0.10 (s, 3H), 0.09 (s, 3H); ^19^F NMR (377 MHz, CDCl_3_): δ −136.63 (d, *J* = 8.6 Hz); ^13^C NMR (101 MHz, CDCl_3_): δ 165.86 (d, *J* = 25.5 Hz), 94.05 (d, *J* = 275.1 Hz), 84.73, 75.13 (d, *J* = 15.4 Hz), 60.12, 25.74, 25.53, 18.23, 18.05, −4.38, −5.18, −5.46, −5.47; HRMS (ESI): *m*/*z* [M + H]^+^ calcd for C_17_H_35_BrFO_4_Si_2_, 457.1241; found, 457.1241.

### (*3S,4S,5R*)-4-((*tert*-Butyldimethylsilyl) oxy)-5-(((*tert*-butyldimethylsilyl)oxy)methyl)-3-chloro-3-fluorotetrahydrofuran-2-ol (**31**)

To 1 g of lactone **26** (2.42 mmol) in 15 mL of toluene at −78 °C was added DIBAL-H (1M in toluene, 3.63 mL) dropwise. After stirring at −78 °C for 3 h, the mixture was quenched by adding MeOH until gas evolution ceased, then poured into 200 mL of ether, and washed with potassium sodium tartrate solution (0.5 M, 200 mL × 2) and water (200 mL × 2). The organic layer was dried over anhydrous sodium sulfate. The solvent was removed to give the product of 0.98 g (98% yield). The ratio of α/β is about 3–4:1. The lactose can be used directly for the next reaction without purification.

Compound **31** was purified by chromatography on silica gel (0 to 66% ethyl acetate in hexane). The conversion was undertaken in an oven at 30, 37, 40, and 45 °C. Temperature was monitored using a thermometer. The ratio of α to β is 5:1 after 4 days at 40 °C. The best ratio of α to β is about 7:1 when the sample was set at 30 °C for 2 weeks.

^1^H NMR (400 MHz, CDCl_3_) of compound **31** at 40 °C for 4 days, δ 5.17 (ddd, *J* = 12.0, 3.2, 0.7 Hz, 4H), 5.05 (dd, *J* = 9.2, 5.6 Hz, 1H), 4.45 (dd, *J* = 17.0, 6.3 Hz, 1H), 4.31 (ddd, *J* = 8.5, 4.6, 0.7 Hz, 5H), 4.03 (tdd, *J* = 5.6, 4.3, 1.7 Hz, 5H), 3.98 (dt, *J* = 6.3, 2.1 Hz, 1H), 3.80–3.61 (m, 12H), 3.45 (dd, *J* = 12.0, 2.3 Hz, 4H), 0.84 (dd, *J* = 6.7, 5.0 Hz, 107H), 0.12 (d, *J* = 8.8 Hz, 16H), 0.09–0.02 (m, 23H), −0.00 (d, *J* = 1.0 Hz, 27H); ^19^F NMR (377 MHz, CDCl_3_): δ −132.98 (d, *J* = 16.6 Hz), −138.57 (d, *J* = 8.6 Hz); HRMS (ESI): *m*/*z* [M + Na]^+^ calcd for C_17_H_36_ClFNaO_4_Si_2_, 437.1722; found, 437.1730.

### 1-((*3S,4S,5R*)-4-((*tert*-Butyldimethylsilyl)oxy)-5-(((*tert*-butyldimethylsilyl)oxy)methyl)-3-chloro-3-fluorotetrahydrofuran-2-yl) Pyrimidine-2,4(1*H*,3*H*)-dione (**33**)

Silylated uracil: A mixture of 5.6 g of uracil (50 mmol), 23 mg of ammonium sulfate, and 56 mL of hexamethyldisilazane (267 mmol) was heated at 125 °C overnight. The excess HMDS was removed under high vacuum at 100 °C. The residue was dissolved in 10 mL of DCM and used for the next coupling reaction. A solution of 0.98 g (2.36 mmol) of compound **31** in 30 mL of DCM was prepared at −78 °C. To the solution was added triflic anhydride (0.55 mL, 3.28 mmol) and DIPEA (0.66 mL, 3.79 mmol) quickly (about 10 s) at this temperature. The mixture was stirred at −78 °C for 1 h. A pre-cooled silylated uracil solution (in step 1) was added using a double-tipped needle at −78 °C. After stirring at −78 °C for 1 h and toward rt for 3 h, the reaction mixture was poured into 200 mL of cold saturated sodium bicarbonate solution, extracted with DCM (200 mL × 2), and dried over Na_2_SO_4_, and the solvent was removed *in vacuo*. The residue was purified by chromatography on silica gel (0–17% ethyl acetate in hexane) to give product **33** (0.98 g, 81%) as a white foam. ^1^H NMR showed that the ratio of α/β is about 1:3.

^1^H NMR (400 MHz, CDCl_3_): δ 7.72 (d, *J* = 8.2 Hz, 2H), 7.40 (dd, *J* = 8.5, 2.9 Hz, 1H), 6.43 (d, *J* = 15.7 Hz, 1H) 6.38 (d, *J* = 15.3 Hz, 2H), 5.78 (dd, *J* = 8.5, 2.0 Hz, 1H), 5.74 (d, *J* = 8.2 Hz, 2H), 4.70 (dd, *J* = 15.8, 7.0 Hz, 1H), 4.37 (dd, 15.6, 8.5 Hz, 2H), 4.17 (m, 1H), 4.05 (dd, *J* = 12.0, 2.0 Hz, 2H), 3.96 (m, *2*H), 3.94 (m, 1H), 3.83 (m, 2H), 3.78 (m, 1H), 0.96, 0.95, 0.94 (s, s, s, 48H), 0.24, 0.18, 0.15, 0.14, 0.11 (s, s, s, s, s); ^13^C NMR (CDCl3) d: 162.3, 162.2, 150.0, 149.8, 140.5, 140.4, 139.0, 113.4 (d, *J* = 255.8 Hz), 109.9 (d, *J* = 261.3 Hz), 102.8, 102.4, 87.7 (d, *J* = 40.6 Hz), 87.1 (d, *J* = 16.5 Hz), 84.3, 81.6, 75.6 (d, *J* = 16.4 Hz), 74.7 (d, *J* = 17.1 Hz), 61.1, 59.9, 25.9, 25.8, 25.6, 18.3, 18.2, 18.0; ^19^F NMR (377 MHz, CDCl_3_): δ −122.95 (t, *J* = 15.8 Hz, β), −138.40 (d, *J* = 15.1 Hz, α); HRMS (ESI): *m*/*z* [M + H]^+^ calcd for C_21_H_39_ClFN_2_O_5_Si_2_, 509.2070; found, 509.2064.

### 4-Amino-1-((*3S,4S,5R*)-4-((*tert*-butyldimethylsilyl) oxy)-5-(((*tert*-butyldimethylsilyl)oxy)methyl)-3-chloro-3-fluorotetrahydrofuran-2-yl) Pyrimidin-2(1*H*)-one (**44**)

By the same procedure described above for **33**, compound **44** can be obtained (α/β = 1:5) in 92% yield from **31** (α/β = 7:1) as a white solid. ^1^H NMR (400 MHz, CDCl_3_): δ 9.81 (br, NH_2_), 7.68 (d, *J* = 7.6 Hz, 1H), 7.61 (d, *J* = 7.6, 1H), 7.35 (dd, *J* = 7.6, 2.5 Hz, 0.3H), 6.40 (d, *J* = 13.6 Hz, 0.3H) 6.31 (d, *J* = 14.8 Hz, 1H), 5.73 (d, *J* = 7.6, 0.3H), 5.70 (d, *J* = 7.6, 1H), 4.85 (d, *J* = 8.4 Hz), 4.53 (dd, *J* = 14.7, 6.8 Hz, 0.2H), 4.21 (dd, 15.3, 8.4 Hz, 1H), 4.04 (m, 0.2H), 3.92(dd, *J* = 11.9, 1.9 Hz, 1H), 3.91 (dd, *J* = 11.9, 0.9 Hz, 1H), 3.78 (dd, *J* = 11.7, 1.8 Hz, 0.3H), 3.68(dd, *J* = 11.8, 1.3 Hz, 1H), 3.63 (dd, *J* = 11.9, 2.5 Hz, 0.3H), 0.82, 0.80, 0.79, 0.78 (s, s, s, s, 29H), 0.08, 0.07, 0.03, 0.02, 0.00, −0.04 (s, s, s, s, s, s, 19H); ^19^F NMR (377 MHz, CDCl_3_): δ −121.6 (t, *J* = 15.1 Hz, β), −136.4 (d, *J* = 14.2 Hz, α); ^13^C NMR (100.65 MHz, CDCl_3_): δ 165.2, 162.3, 160.1, 156.9, 156.4, 156.2, 142.3, 140.8, 123.5 (d), 113.5 (d), 95.6, 95.3, 89.5, 88.1 (d), 84.5, 81.6, 76.0, 74.9 (d), 61.2, 60.0, 25.9, 25.8, 25.6, 25.5, 18.3, 18.2, 18.0, −4.1, −4.3, −5.0, −5.1; HRMS (ESI): *m*/*z* [M + H]^+^ calcd for C_21_H_40_ClFN_3_O_4_Si_2_, 508.2230; found, 508.2224.

### (*2S,3S,4R,5R*)-3-Bromo-4-((*tert*-butyldimethylsilyl)oxy)-5-(((tert-butyldimethylsilyl)oxy)methyl)-3-fluorotetrahydrofuran-2-ol (**32**)

Compound **40** was reduced with LiAl(OBu)_3_H as above for **31**. After workup and purification by silica gel column chromatography (0–10% EtOAc/hexane), pure **32** was obtained in 97% as a syrup.

5 g of lactol **32** was then heated at 45 °C in a temperature-controlled oven, and a solid product **32** was obtained after about a week. α/β > 12 is based on ^1^H NMR. ^1^H NMR (400 MHz, CDCl_3_): δ 5.38 (dd, *J* = 12.0, 3.8, 0.93H), 5.18 (dd, *J* = 9.1, 5.5 Hz, 0.07H), 4.65 (dd, *J* = 17.9, 6.1 Hz, 0.07H), 4.55 (dd, *J* = 9.7, 4.8 Hz, 0.93H), 4.10 (ddd, *J* = 10.2, 5.2, 1.4 Hz, 1H), 3.85 (dd, *J* = 11.2, 5.3 Hz, 1H), 3.76 (dd, 11.2, 3.8 Hz, 1H), 3.56–3.51 (m, 1H), 0.92 (s, 9H), 0.91 (s, 9H), 0.21 (s, 3H), 0.16 (s, 3H), 0.09 (s, 3H), 0.08 (s, 3H); ^19^F NMR (377 MHz, CDCl_3_): δ −131.12, −138.00; ^13^C NMR (100.65 MHz, CDCl_3_): δ 101.61 (d, *J* = 274.8 Hz), 101.20 (d, *J* = 19.1 Hz), 83.84, 61.99, 25.86, 25.60, 18.32, 17.99, −4.45, −5.05, −5.36, −5.43; HRMS (ESI): *m*/*z* [M + Na]^+^ calcd for C_17_H_36_BrFNaO_4_Si_2_, 481.1217; found, 481.1213.

### 1-((*2R,3S,4R,5R*)-3-Bromo-4-((*tert*-butyldimethylsilyl)oxy)-5-(((*tert*-butyldimethyl silyl)oxy)methyl)-3-fluorotetrahydrofuran-2-yl) Pyrimidine-2,4(1*H*,3*H*)-dione (**34**)

Step 1. A mixture of 28 g of uracil, 280 mL of HMDS, and 112 mg of ammonium sulfate was stirred at 125 °C to form a clear solution (normally about 3 h). The excess HMDS was removed under high vacuum (∼5–10 mmHg) at 80–100 °C in an oil bath. The residue was dissolved in 200 mL of DCM and pre-cooled at −78 °C for step 2.

Step 2. A solution of lactol **32** (5 g, α/β (^1^H NMR), 12/1) in DCM (30 mL) was prepared at −78 °C. To this solution was quickly added triflic anhydride (2.8 mL) and 3.3 mL of DIPEA (∼10 s) at −78 °C. The reaction mixture was stirred at −78 °C for 50 min. A pre-cooled silylated uracil solution (in step 1) was added using a double-tipped needle at −78 °C. The reaction mixture was stirred at −78 °C for 1 h, which was then increased to rt, and the mixture was stirred at rt for 4 h (Rf: 0.1, EtOAc/hex, 3/20). The reaction mixture was cooled with an ice bath and was quenched by addition of sat. NaHCO_3_ (10 mL). The solid was filtered through a celite pad, and the celite pad was washed with DCM (2 × 80 mL). The combined filtrates were separated, and the organic layer was washed with sat. NaHCO_3_ and brine and dried over sodium sulfate (TLC: one spot, 20% EtOAc/hexane). After removing the solvent, the residue was purified using a silica column (0 to 25% EtOAc/hexane) to give 5.12 g of product **34** in 85% yield (β/α, 5.2:1 based on ^1^H NMR).

^1^H NMR (400 MHz, CD_3_OD): δ 7.71 (d, *J* = 8.2 Hz, 0.83H), 7.63 (dd, *J* = 8.2, 3.2 Hz, 0.18H), 6.52 (d, *J* = 16.6 Hz, 0.16H) 6.35 (d, *J* = 16.4 Hz, 0.82H), 5.74 (d, *J* = 8.2 Hz, 0.16H), 5.71 (d, *J* = 8.2 Hz, 0.84H), 4.55 (dd, *J* = 17.4, 8.3 Hz, 1H), 4.12 (dd, *J* = 12.2, 1.9 Hz, 1H), 4.01–3.96 (m, 1H), 3.87 (dd, *J* = 12.2, 2.1 Hz, 1H), 0.96, 0.95, 0.94, 0.93 (s, s, s, s, 18H), 0.28, 0.27 (s, s, 3H), 0.22, 0.21 (s, s, 3H), 0.16, 0.15, 0.12, 0.11 (s, s, s, s, 6H); ^19^F NMR (377 MHz, CD_3_OD): δ −119.8 (β), −137.8 (α); ^13^C NMR (100.66 MHz, CD_3_OD): δ 165.60, 165.34, 152.11, 151.86, 142.72, 140.52, 110.4 (d, *J* = 265.9 Hz), 103.46, 103.04, 89.82 (d, *J* = 40.0 Hz), 89.02 (d, *J* = 15.4 Hz), 85.09, 83.04, 78.21 (d, *J* = 15.5 Hz), 77.63 (d, *J* = 16.3 Hz), 62.19, 61.24, 26.45, 26.33, 26.17, 26.14, 19.26, 18.94, −3.58, −3.80, −4.36, −4.54, −5.23, −5.30, −5.33; HRMS (ESI): *m*/*z* [M + H]^+^ calcd for C_21_H_39_BrFN_2_O_5_Si_2_, 553.1565; found, 553.1556.

### 4-Amino-1-((*2R,3S,4R,5R*)-3-bromo-4-((*tert*-butyldimethylsilyl)oxy)-5-(((*tert*-butyldimethylsilyl)oxy)methyl)-3-fluorotetrahydrofuran-2-yl) Pyrimidin-2(1*H*)-one (**43**)

A similar procedure was employed for preparing nucleoside **34** using lactol **32** in 80% yield (β/α, 5:1 based on ^1^H NMR).

^1^H NMR (400 MHz, CD_3_OD): δ 7.72 (d, *J* = 7.6 Hz, 0.83H), 7.59 (dd, *J* = 7.6, 3.2 Hz, 0.17H), 6.66 (d, *J* = 16.8 Hz, 0.17H), 6.43 (d, *J* = 16.8 Hz, 0.83H), 4.54 (dd, *J* = 17.6, 8.8 Hz, 1H), 4.13–3.85 (m, 3H), 0.97 (s, 9H), 0.95 (s, 9H), 0.27 (s, 3H), 0.21 (s, 3H), 0.16 (s, 3H), 0.15 (s, 3H); ^19^F NMR (377 MHz, CD_3_OD): δ −121.15, −139.06 (t, *J* = 17.3 Hz); ^13^C NMR (101 MHz, CD_3_OD): δ 167.32, 157.58 (d, *J* = 5.1 Hz), 141.18, 110.63 (d, *J* = 267.1 Hz), 104.57 (d, *J* = 273.8 Hz), 96.72, 96.34, 90.23 (d, *J* = 39.4 Hz), 89.52 (d, *J* = 15.1 Hz), 84.92, 82.75, 78.36 (d, *J* = 15.8 Hz), 77.63 (d, *J* = 16.5 Hz), 62.22, 61.20, 30.75, 26.58, 26.46, 26.35, 26.27, 26.19, 26.16, 19.22, 19.10, 18.93, −3.56, −3.80, −4.36, −4.54, −5.18, −5.23, −5.30, −5.32; HRMS (ESI): *m*/*z* [M + H]^+^ calcd for C_21_H_40_BrFN_3_O_4_Si_2_, 552.1725; found, 552.1717.

### (2*S*)-2′-α-Fluoro-2′-β-bromo-deoxyuridine (**14**)

To a solution of *bis*-TBS-protected-nucleoside **34** (6.2 g, 11.23 mmol) in THF (80 mL) was added 3HF·NEt_3_ (9.3 mL, 56.5 mmol). After stirring for 16 h at rt, the reaction mixture was filtrated on a short silica gel column and washed with 30% MeOH in DCM. After removal of the solvent *in vacuo*, the crude was purified by flash column chromatography using CH_2_Cl_2_/MeOH 9:1 as an eluent to afford the deprotected nucleoside mixture (3.6 g, 99% yield, β/α: 5.2/1) as a white solid. The nucleoside was dissolved in about 14 mL of *i*-PrOH/H_2_O (98:2) under reflux conditions. The solution was cooled to rt, and the formed crystal was collected after 1 day, yielding 1.9 g of pure beta-isomer nucleoside **14**. The mother liquid was removed from solvents, and the residue was purified by flash column chromatography using toluene/acetone to afford the second aliquot of **14** (0.5 g). The total yield was 66.7%. Nucleoside **14** was confirmed by NMR and single-crystal X-ray analysis (single crystals obtained from EtOH). ^1^H NMR (400 MHz, CD_3_OD): δ 7.94 (d, *J* = 8.2 Hz, 1H), 6.33 (d, *J* = 16.4 Hz, 1H), 5.75 (d, *J* = 8.2 Hz, 1H), 4.42 (dd, *J* = 19.7, 9.2 Hz, 1H), 4.01–3.91 (m, 2H), 3.78 (dd, *J* = 12.7, 2.6 Hz, 1H); ^19^F NMR (377 MHz, CD_3_OD): δ −121.17; ^13^C NMR (101 MHz, CD_3_OD): δ 165.64, 152.04, 141.33, 110.69 (d, *J* = 262.4 Hz), 103.25, 89.82 (d, *J* = 39.9 Hz), 82.78, 76.60 (d, *J* = 17.2 Hz), 59.88; HRMS (ESI): *m*/*z* [M + H]^+^ calcd for C_9_H_10_BrFN_2_O_5_, 324.9835; found, 324.9825.

## Supporting Information Available

Copies of ^1^H, ^13^C, and ^19^F NMR spectra (PDF)
