# Supplementary material for: Diastereoselective Synthesis of 2′-Dihalopyrimidine Ribonucleoside Inhibitors of Hepatitis C Virus Replication
Source: ACS Omega. 2021 Dec 22;7(1):1452–61. doi: 10.1021/acsomega.1c06174 (PMC8756791; doi:10.1021/acsomega.1c06174)
Supplement: Supplementary file 1 — ao1c06174_si_001.pdf [file ao1c06174_si_001.pdf]

Supporting Information

Diastereoselective Synthesis of 2'-Dihalo-Pyrimidine Ribonucleoside Inhibitors of Hepatitis C Virus Replication

Longhu Zhou, Hongwang Zhang, Chengwei Li, Coralie De Schutter, Ozkan Sari, Seema Mengshetti, Shaoman Zhou, Mahesh Kasthuri, Steven J. Coats, Raymond F. Schinazi* and Franck Amblard**

Center for AIDS Research, Laboratory of Biochemical Pharmacology, Department of Pediatrics, Emory University School of Medicine, and Children's Healthcare of Atlanta, 1760 Haygood Drive, Atlanta, GA 30322, USA

*Email: rschina@emory.edu; famblar@emory.edu

Table of contents

^{1}H, ^{13}C and ^{19}F NMR Spectra 2-19

3. ^{1}H, ^{13}C and ^{19}F NMR Spectra

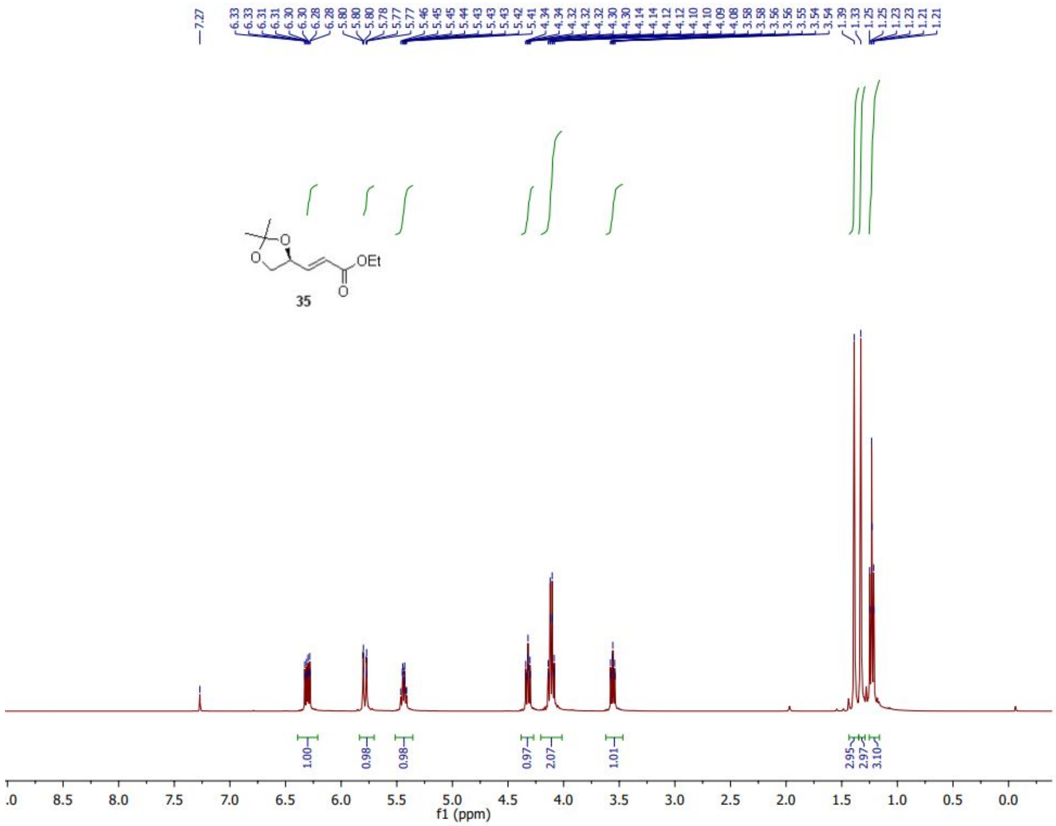

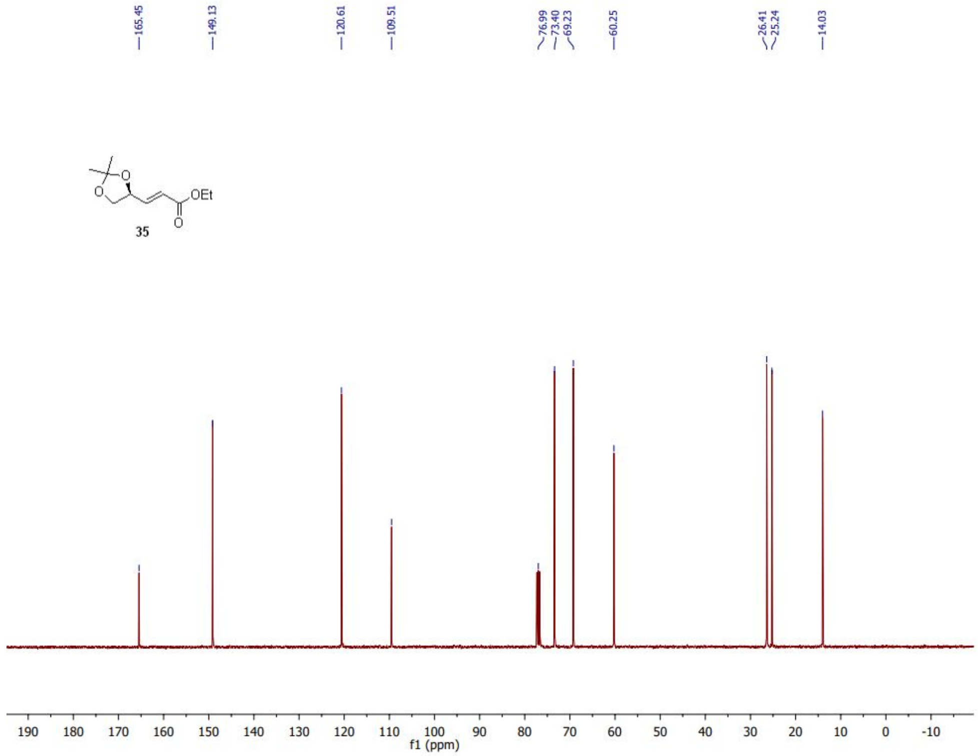

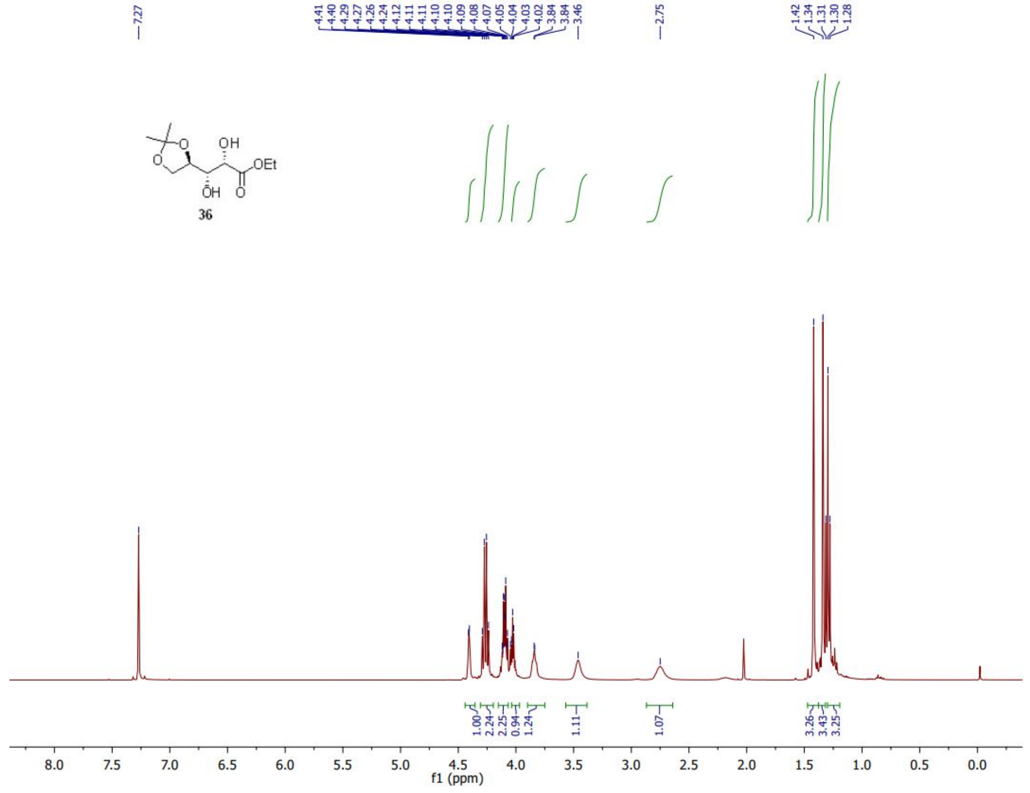

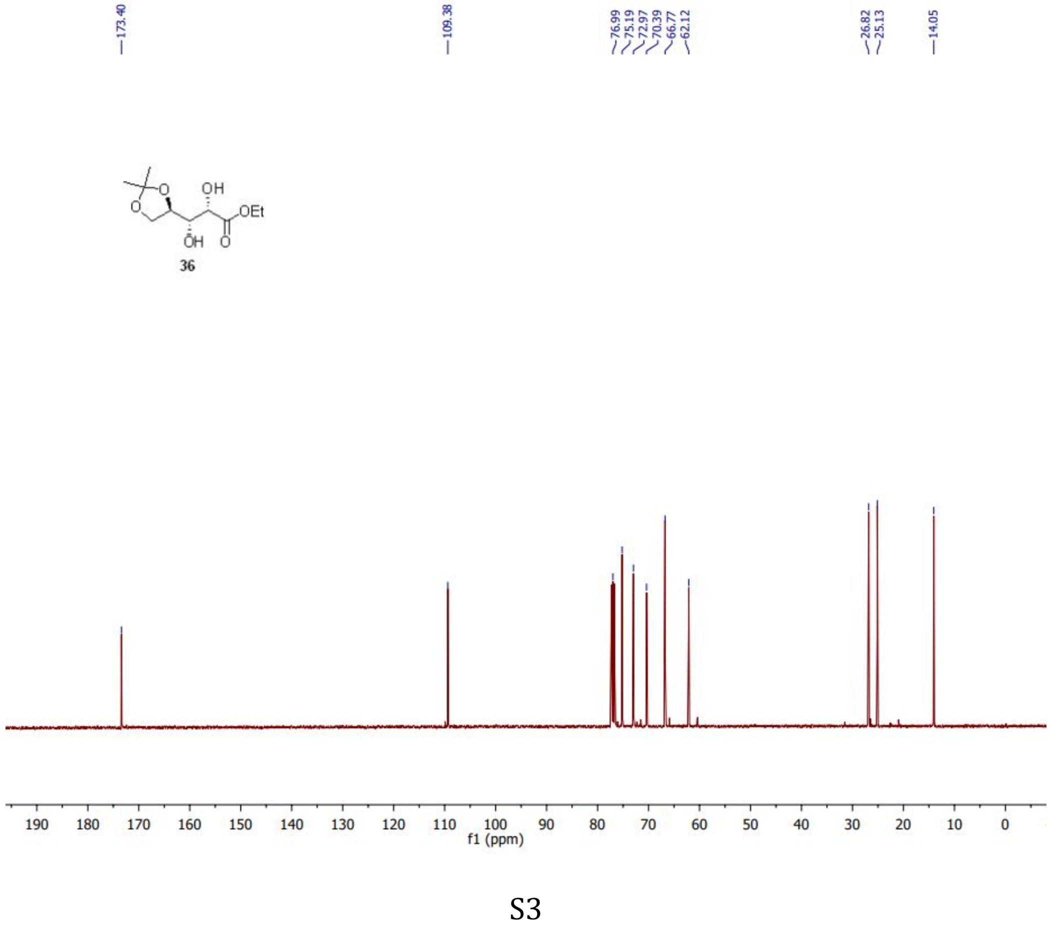

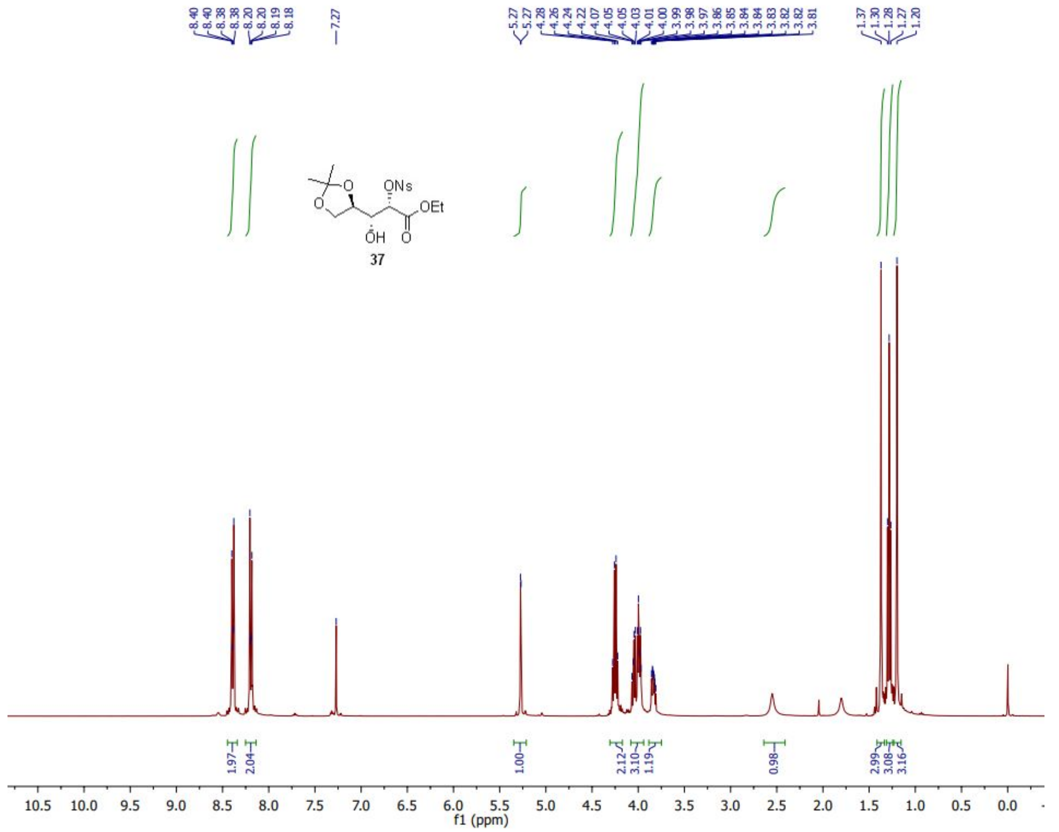

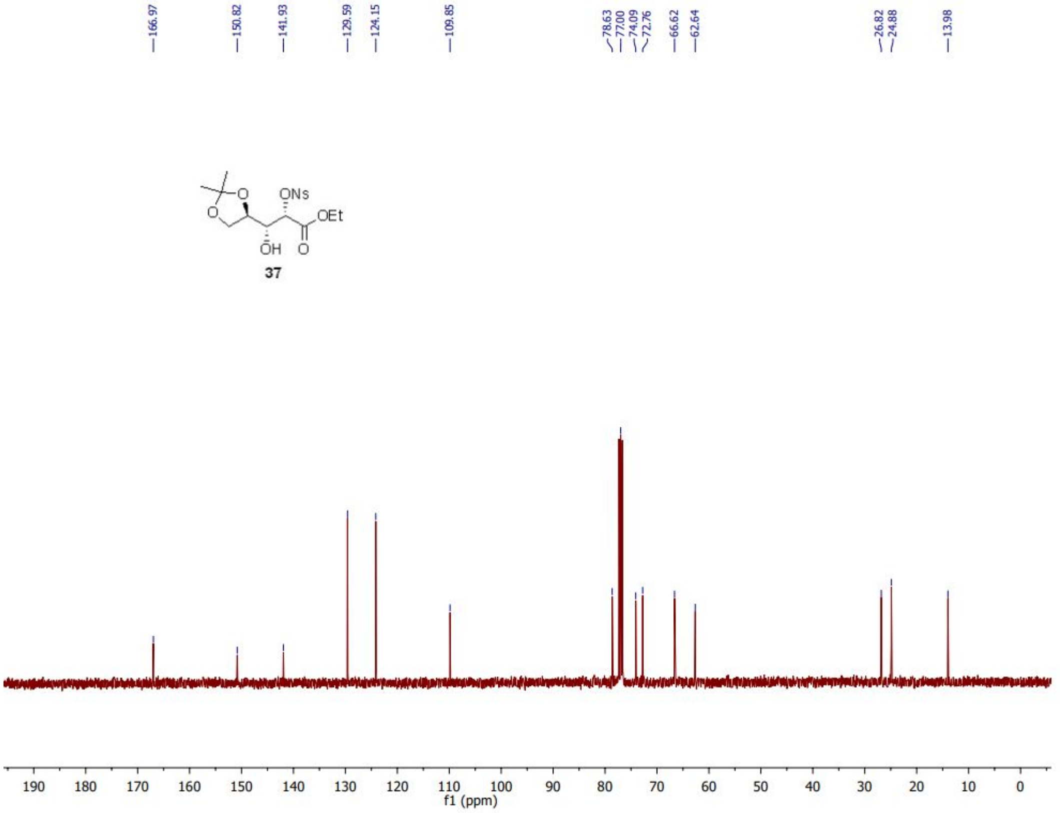

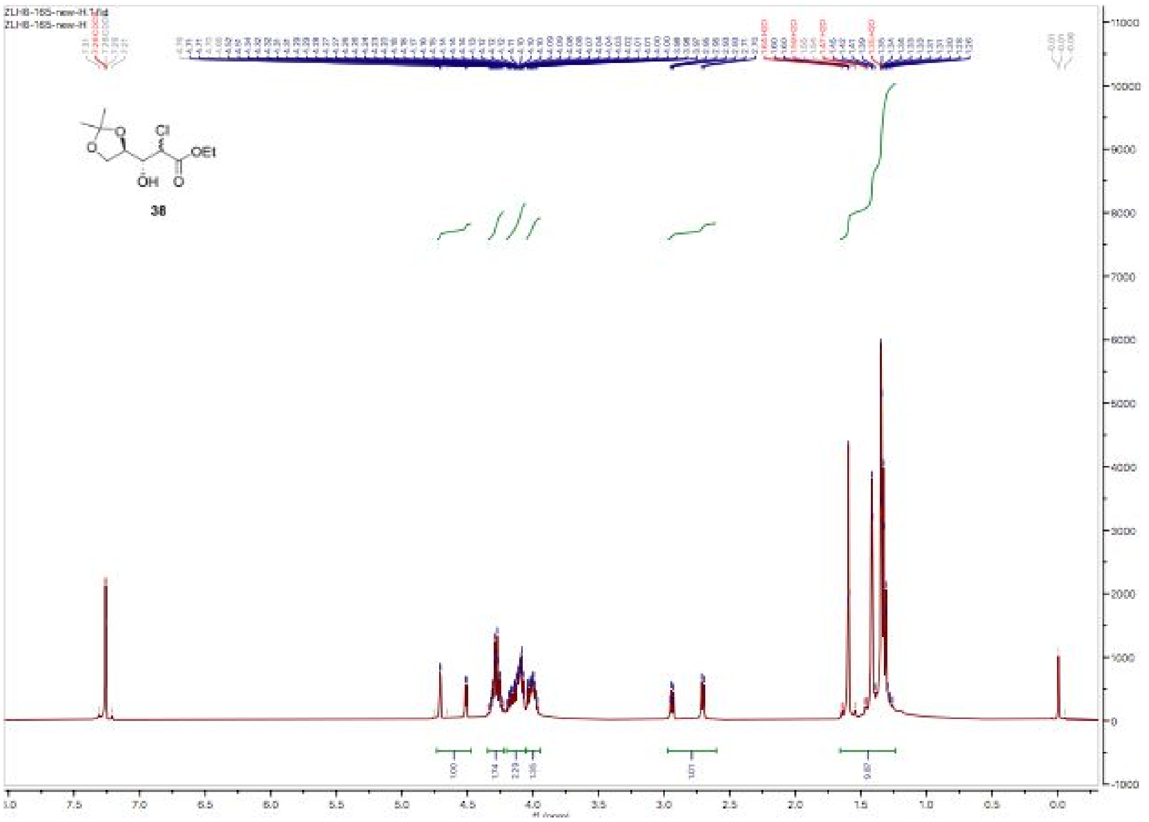

38

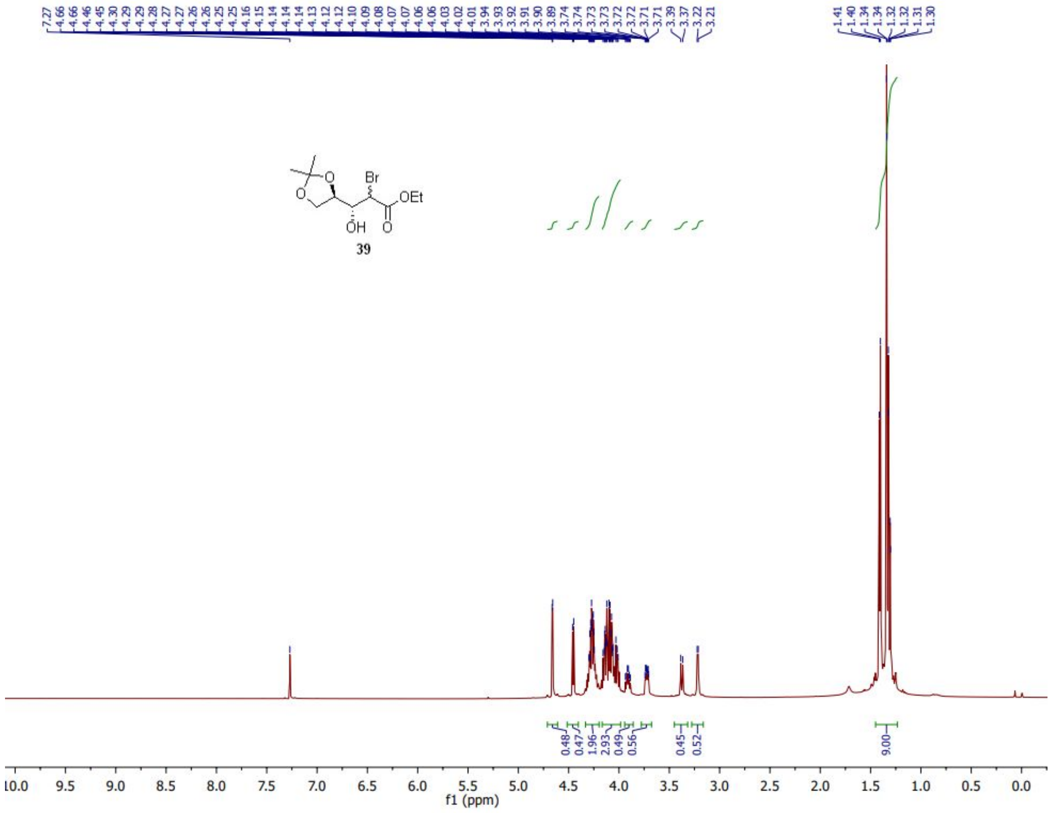

Br
OEt
OH
39
f1 (ppm)

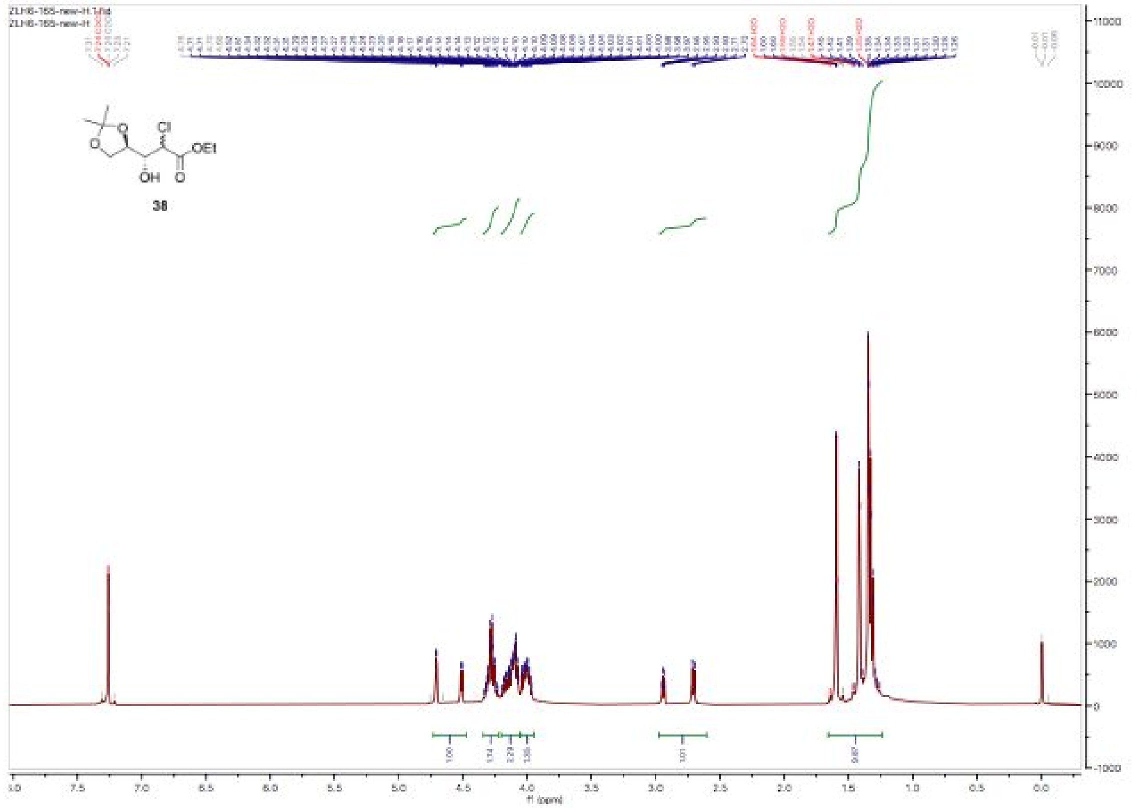
ZLH6-165-new-H.1.fid
38
OEt
Cl
OH
f1 (ppm)

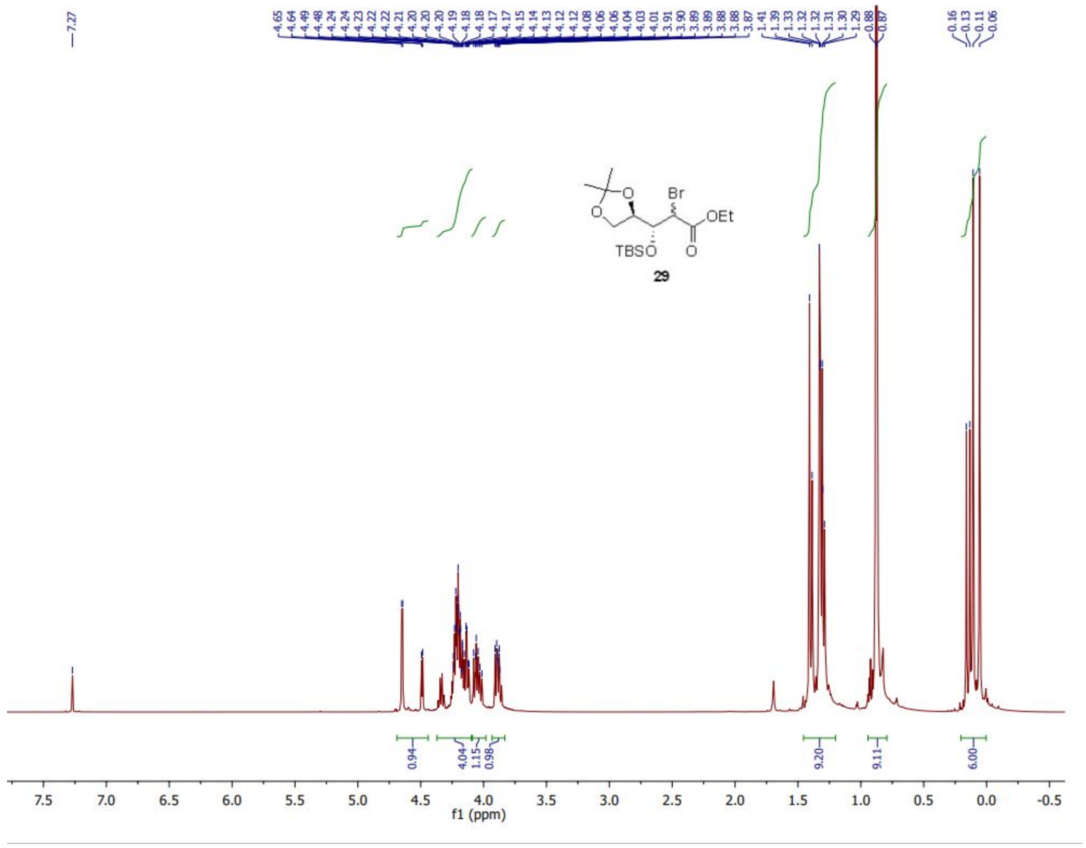
Br
OEt
TBSO
29
f1 (ppm)

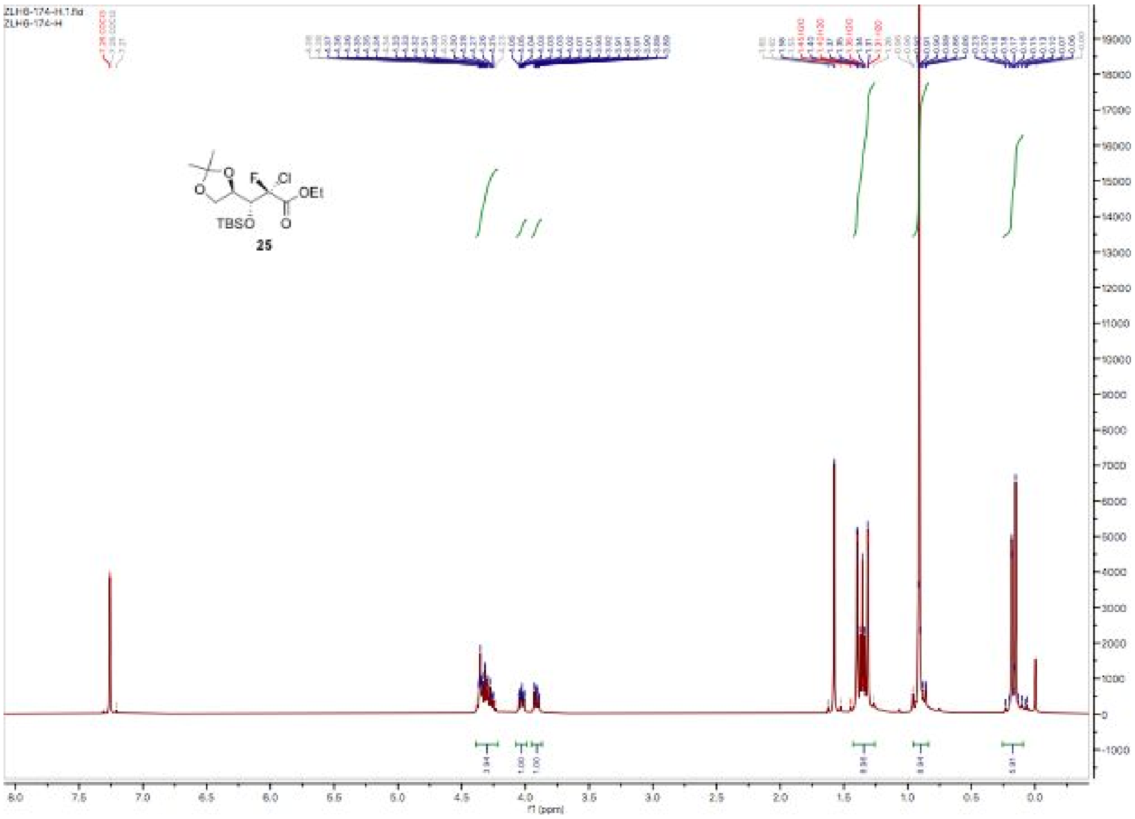

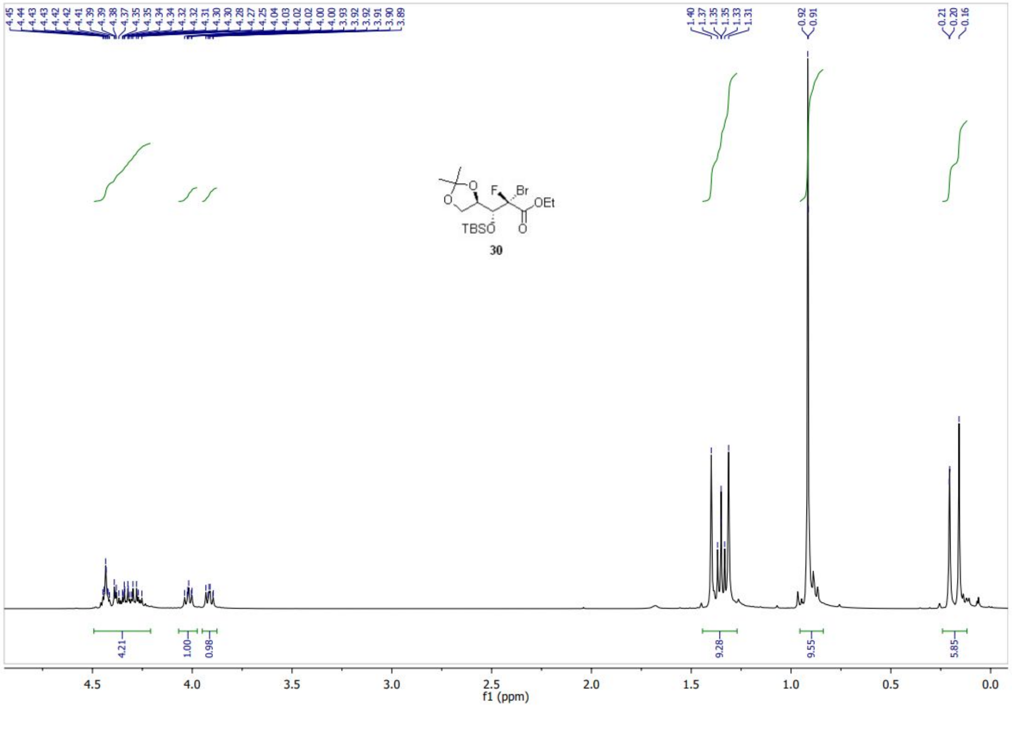

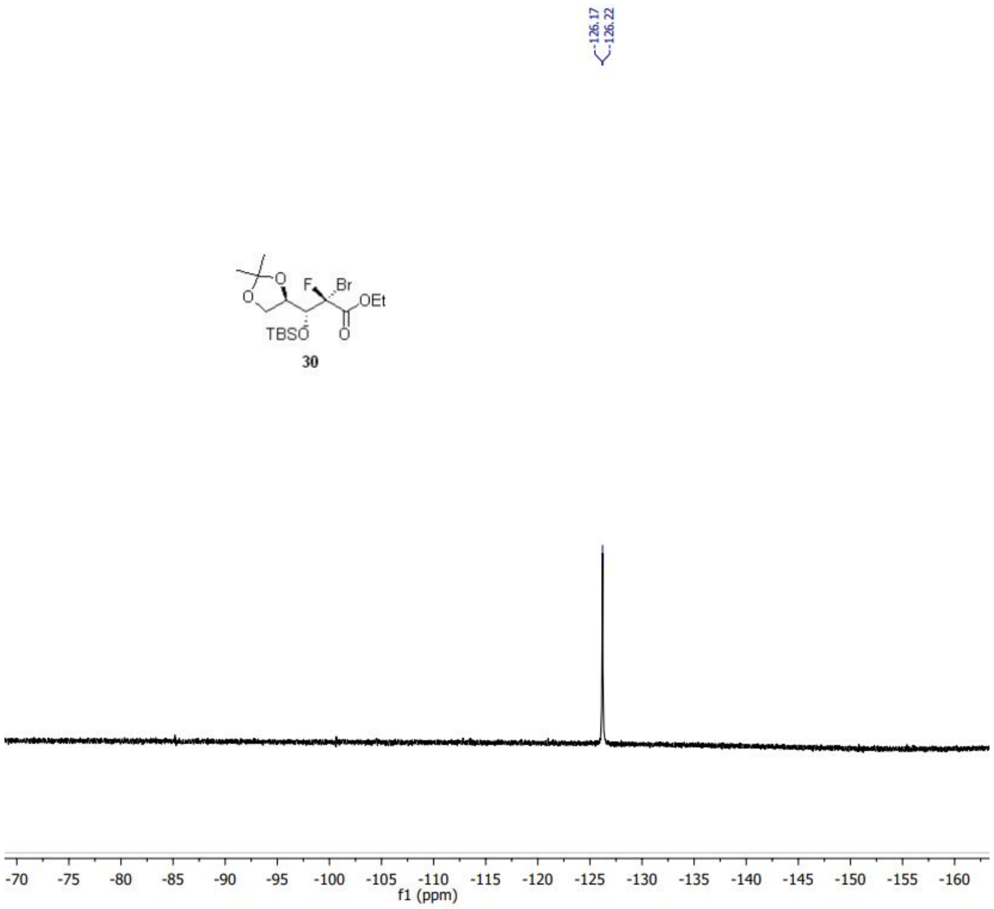
-126.17
-126.22
F
Br
OEt
O
O
O
TBSO
30
-70 -75 -80 -85 -90 -95 -100 -105 -110 -115 -120 -125 -130 -135 -140 -145 -150 -155 -160
f1 (ppm)

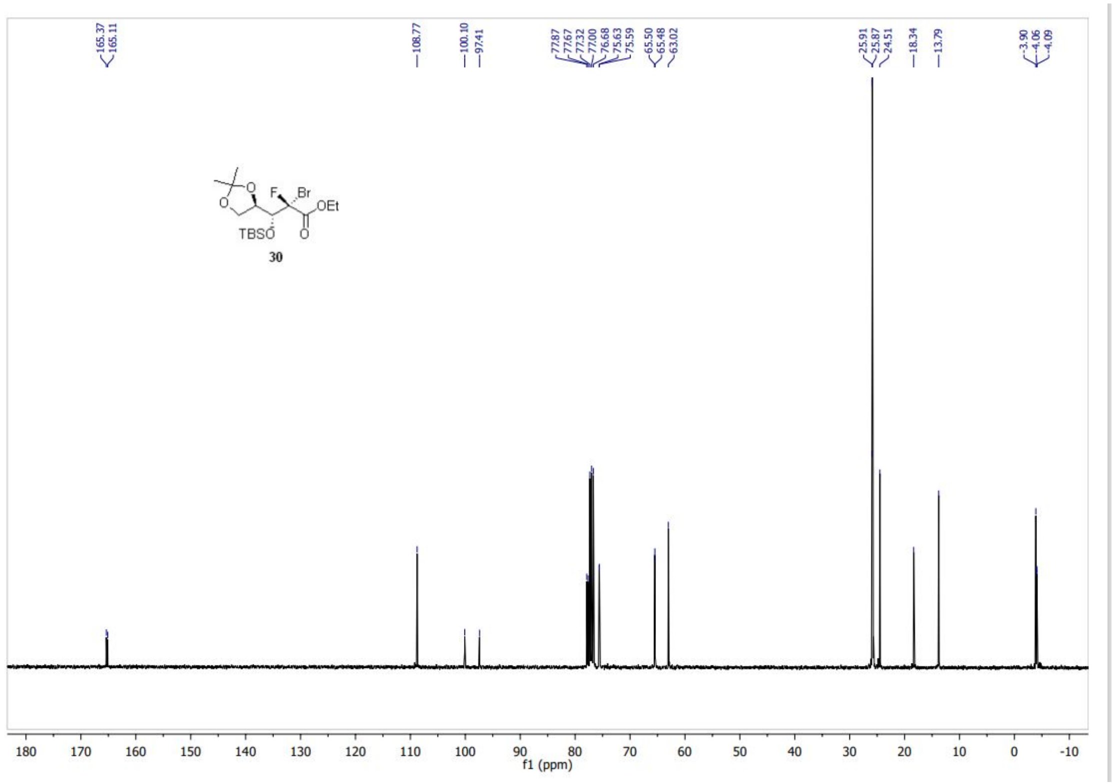
165.37
165.11
108.77
100.10
97.41
77.87
77.67
77.32
77.00
76.68
75.63
75.59
65.50
65.48
63.02
25.91
25.87
24.51
18.34
13.79
-3.90
-4.06
-4.09
F
Br
OEt
O
O
O
TBSO
30
180 170 160 150 140 130 120 110 100 90 80 70 60 50 40 30 20 10 0 -10
f1 (ppm)

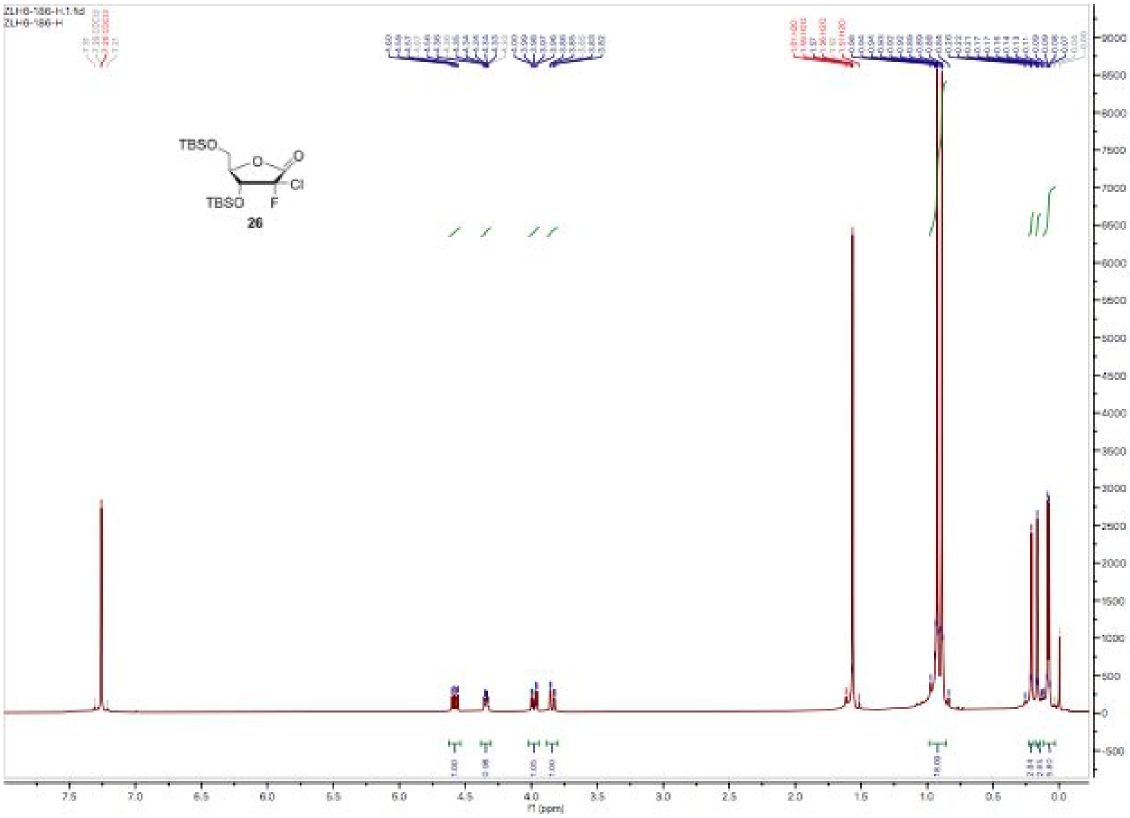

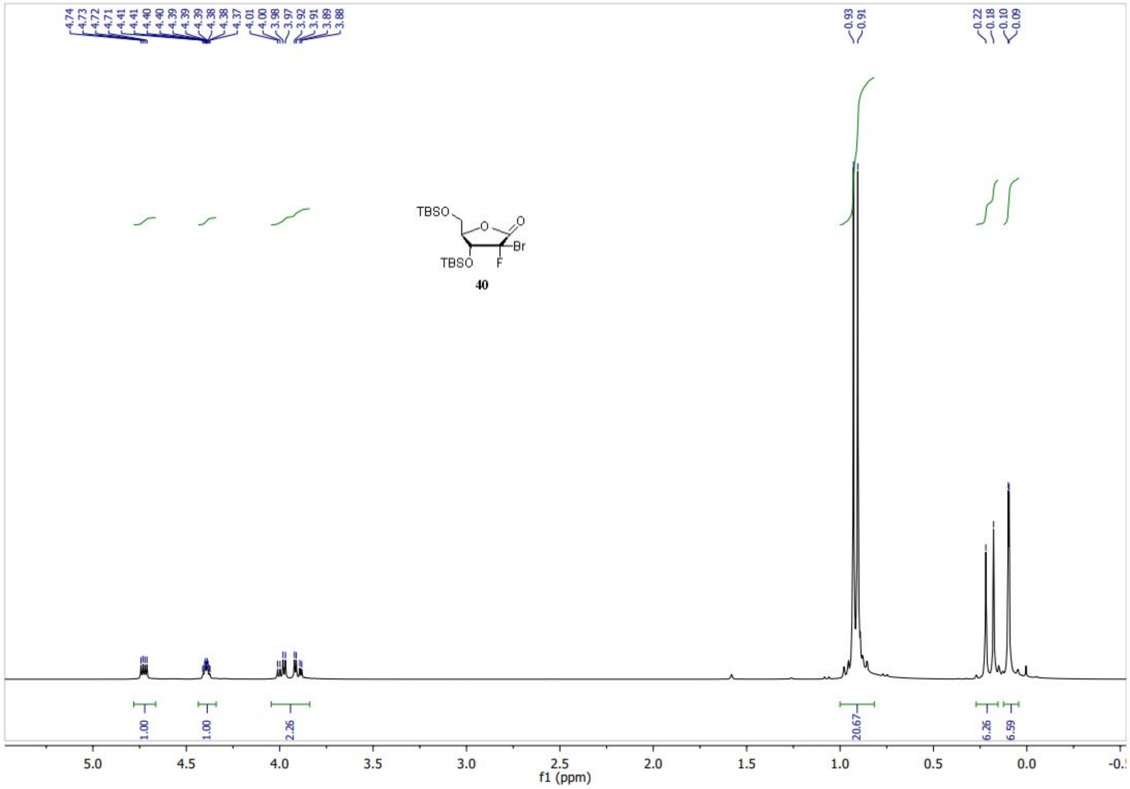

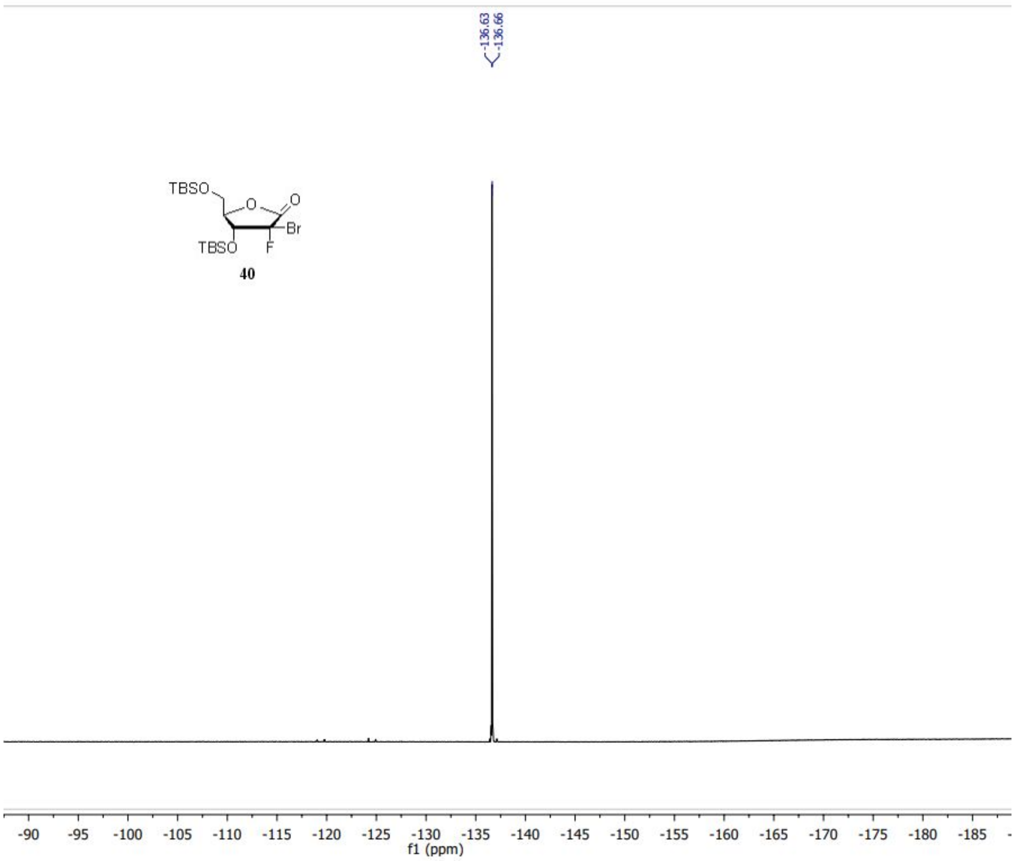
-136.63
-136.66
TBSO
O
O
Br
TBSO
F
40
-90 -95 -100 -105 -110 -115 -120 -125 -130 -135 -140 -145 -150 -155 -160 -165 -170 -175 -180 -185
f1 (ppm)

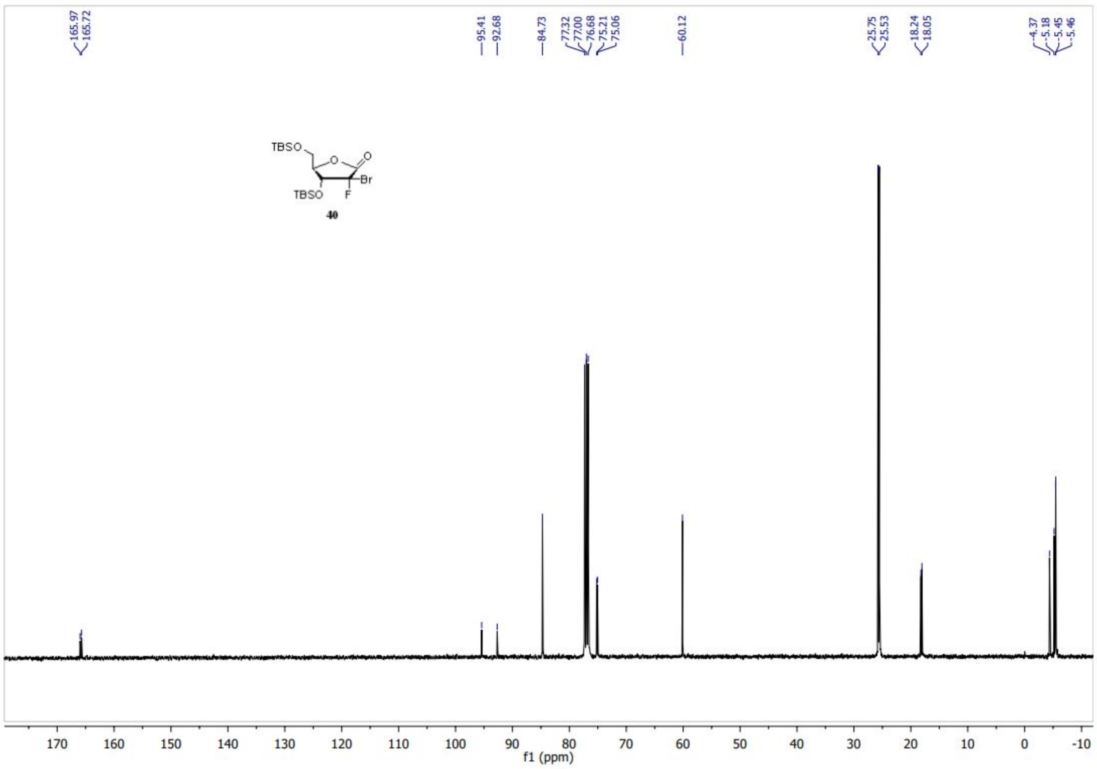
165.97
165.72
95.41
92.68
84.73
77.32
77.00
76.68
75.21
75.06
60.12
25.75
25.53
18.24
18.05
-4.37
-5.18
-5.45
-5.46
TBSO
O
O
Br
TBSO
F
40
170 160 150 140 130 120 110 100 90 80 70 60 50 40 30 20 10 0 -10
f1 (ppm)

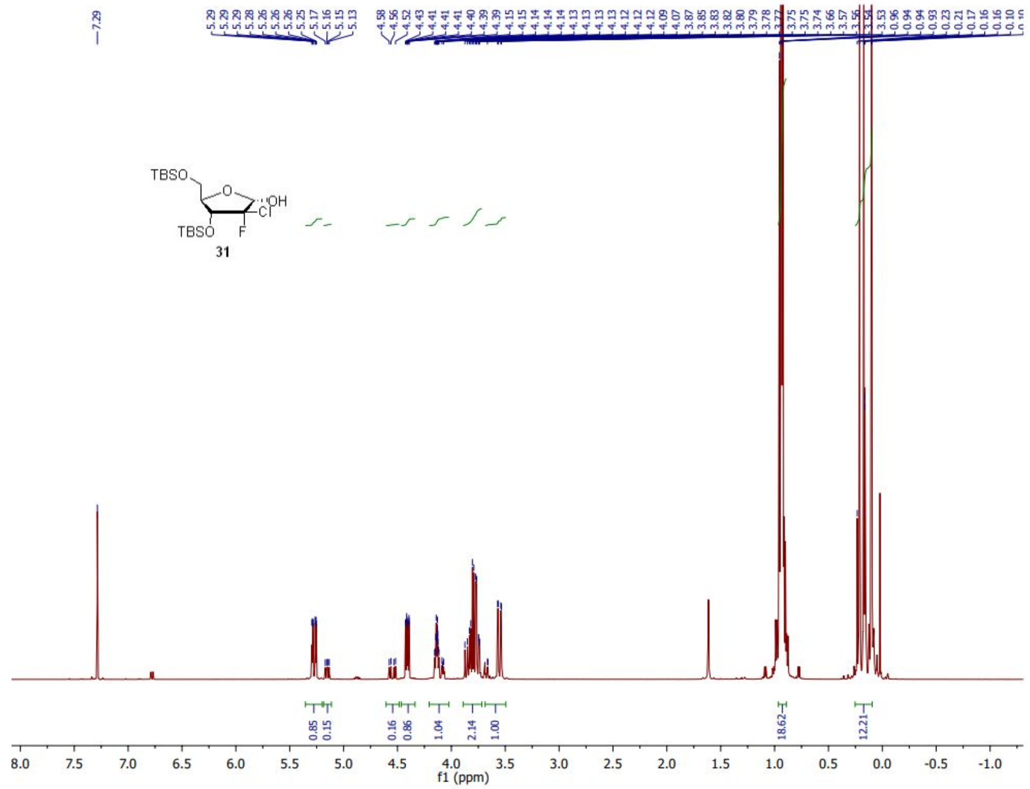
TBSO
OH
Cl
TBSO
F
31
0.85
0.15
0.16
0.86
1.04
2.14
1.00
18.62
12.21
8.0 7.5 7.0 6.5 6.0 5.5 5.0 4.5 4.0 3.5 3.0 2.5 2.0 1.5 1.0 0.5 0.0 -0.5 -1.0
f1 (ppm)

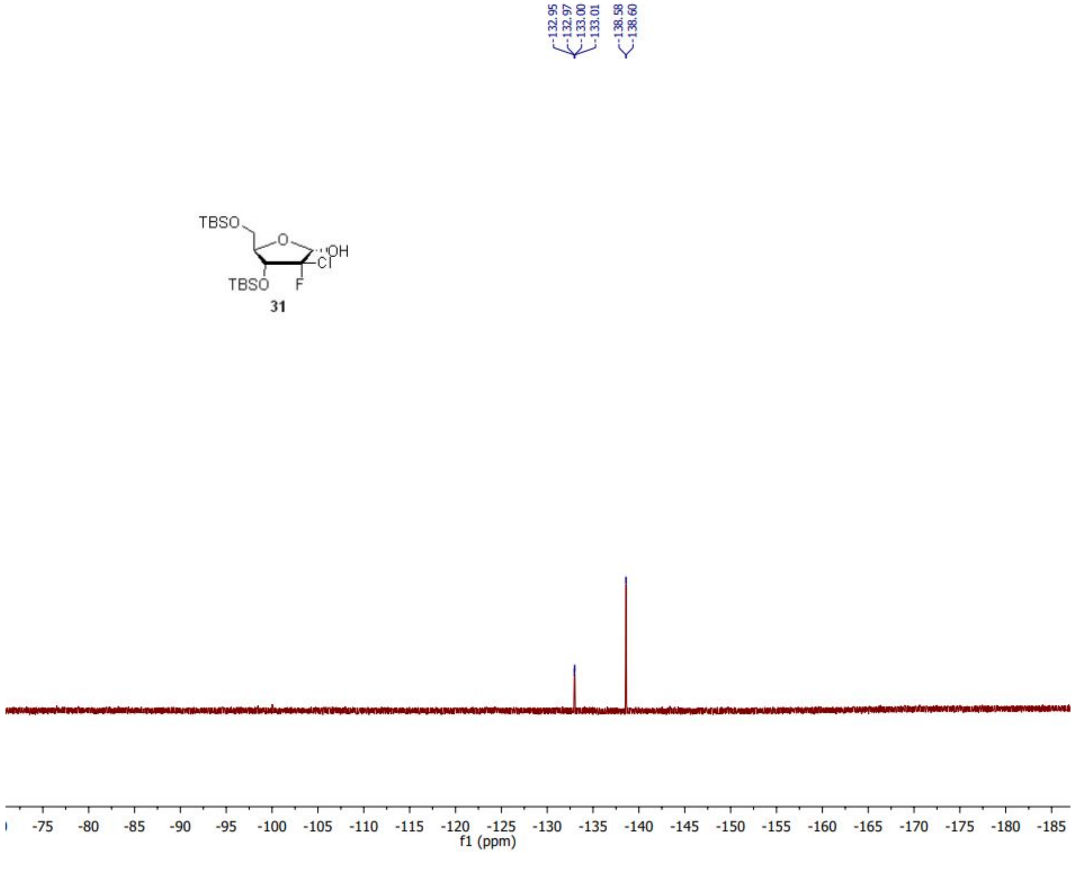
-132.95
-132.97
-133.00
-133.01
-138.58
-138.60
TBSO
OH
Cl
TBSO
F
31
-75 -80 -85 -90 -95 -100 -105 -110 -115 -120 -125 -130 -135 -140 -145 -150 -155 -160 -165 -170 -175 -180 -185
f1 (ppm)

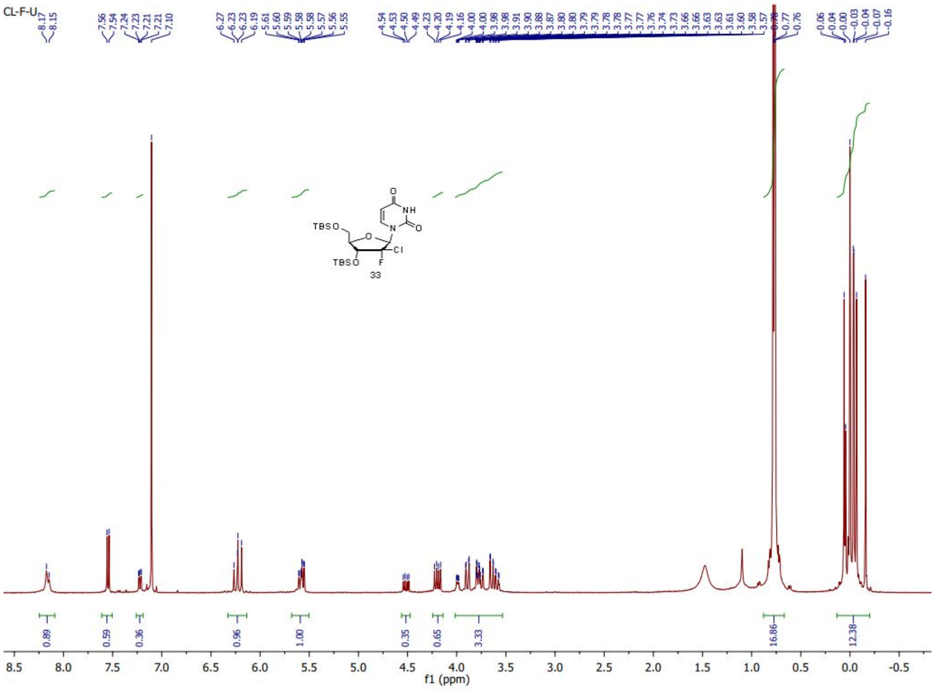
CL-F-U
TBSO
TBSO
NH
N
O
O
O
F
Cl
f1 (ppm)

33

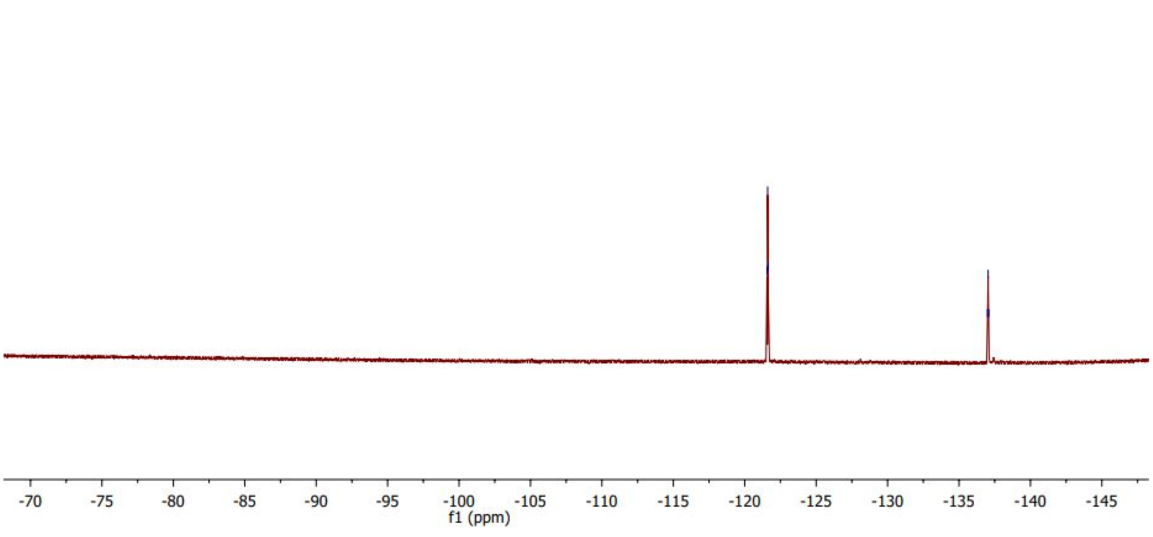
-121.56
-121.60
-121.64
-137.00
-137.04
-137.08
f1 (ppm)

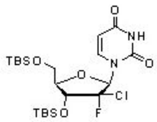
TBSO
TBSO
NH
N
O
O
O
F
Cl

33

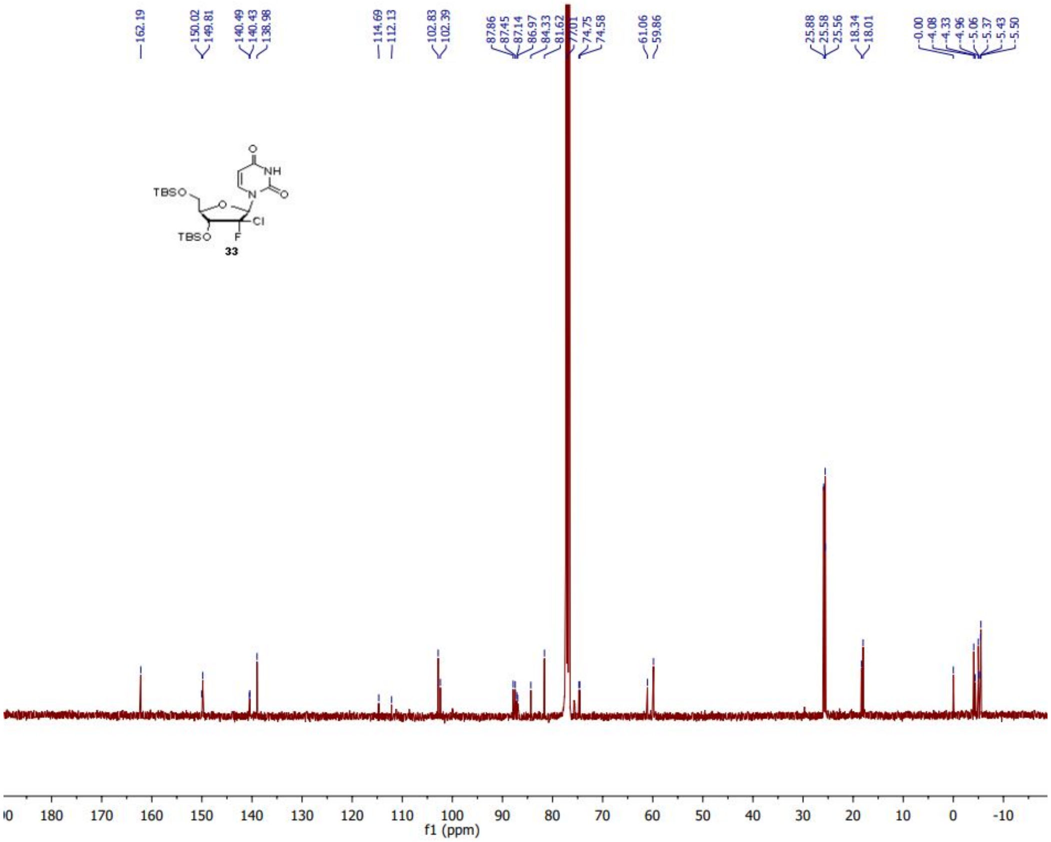
162.19
150.02 149.81
140.49 140.43 138.98
114.69 112.13
102.83 102.39
87.86 87.45 87.14 86.97 84.33 81.62 77.01 74.75 74.58
61.06 59.86
25.88 25.58 25.56 18.34 18.01
-0.00 -4.08 -4.33 -4.96 -5.06 -5.37 -5.43 -5.50
TBSO
NH
O
Cl
F
33
f1 (ppm)

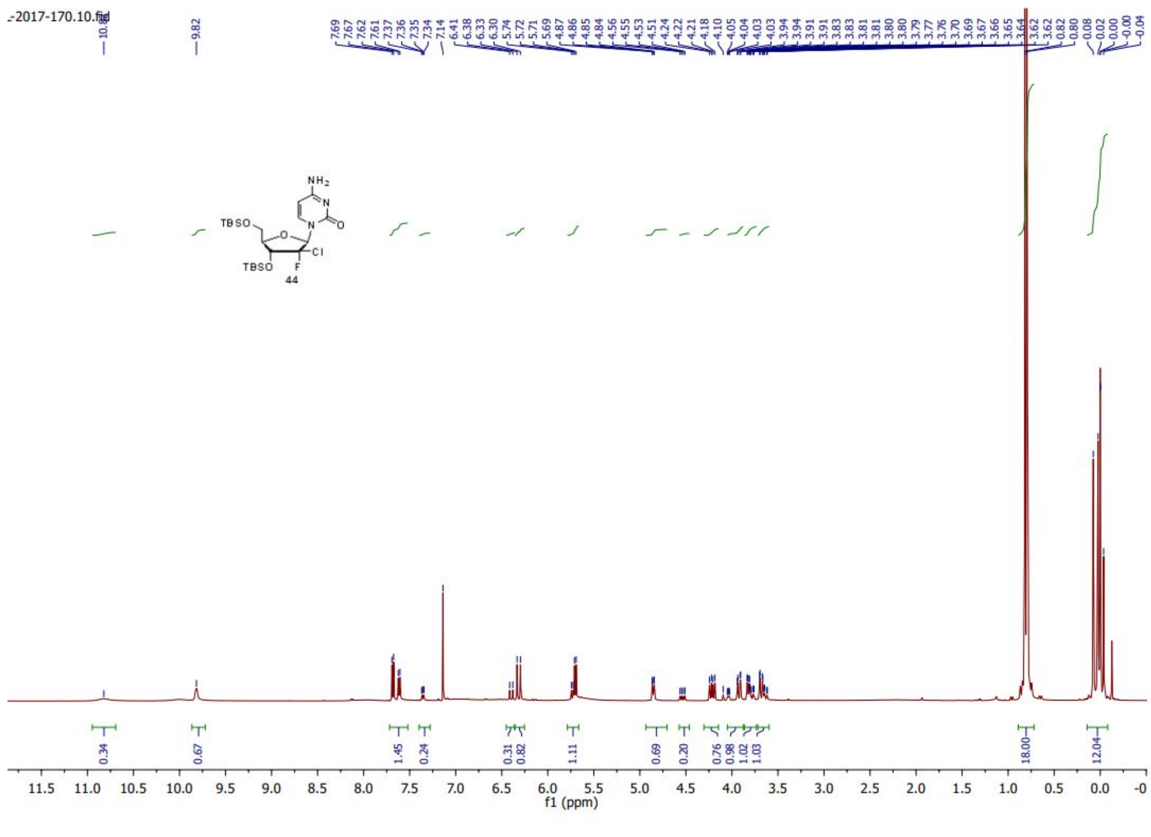
-2017-170.10.fid
9.82
7.69 7.67 7.62 7.61 7.37 7.36 7.35 7.34 7.14 6.41 6.38 6.33 6.30 5.74 5.72 5.71 5.69 4.87 4.86 4.85 4.84 4.56 4.55 4.53 4.51 4.24 4.22 4.21 4.18 4.10 4.05 4.04 4.03 4.03 3.94 3.94 3.91 3.83 3.83 3.81 3.80 3.79 3.77 3.76 3.70 3.69 3.67 3.66 3.65 3.64 3.62 0.82 0.80 0.08 0.02 0.00 -0.00 -0.04
NH_2
TBSO
N
O
Cl
F
44
0.34 0.67 1.45 0.24 0.31 0.82 1.11 0.69 0.20 0.76 0.98 1.02 1.03 18.00 12.04
f1 (ppm)

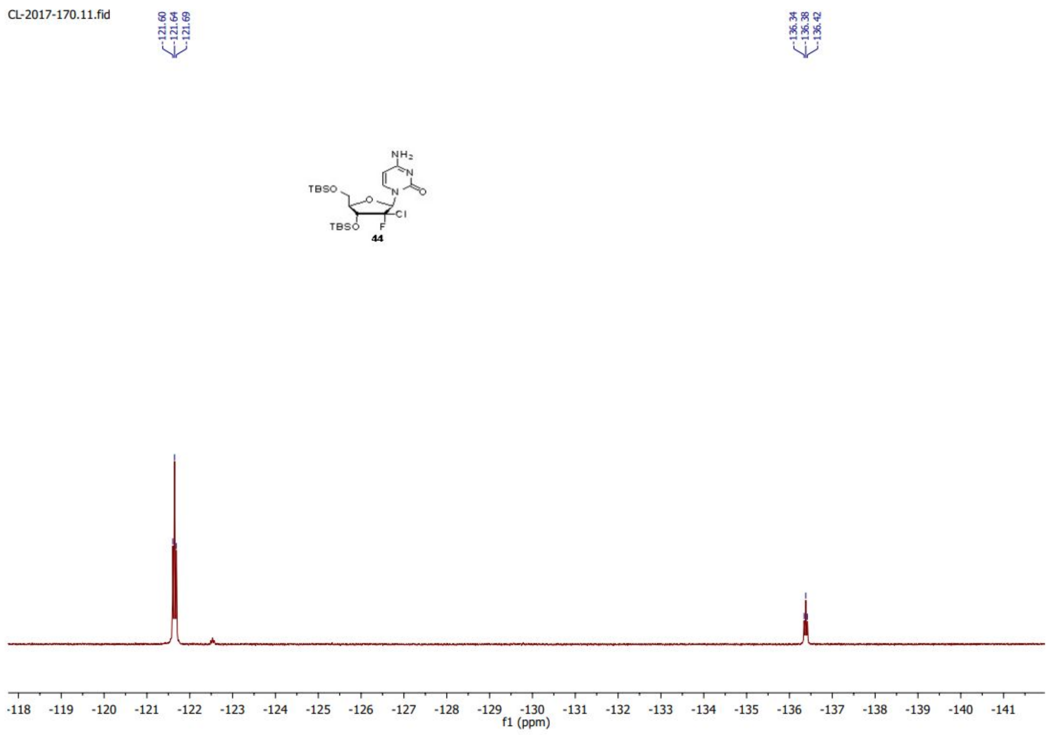
CL-2017-170.11.fid
-121.60
-121.64
-121.69
-136.34
-136.38
-136.42
NH2
TBSO
TBSO
F
Cl
44
f1 (ppm)

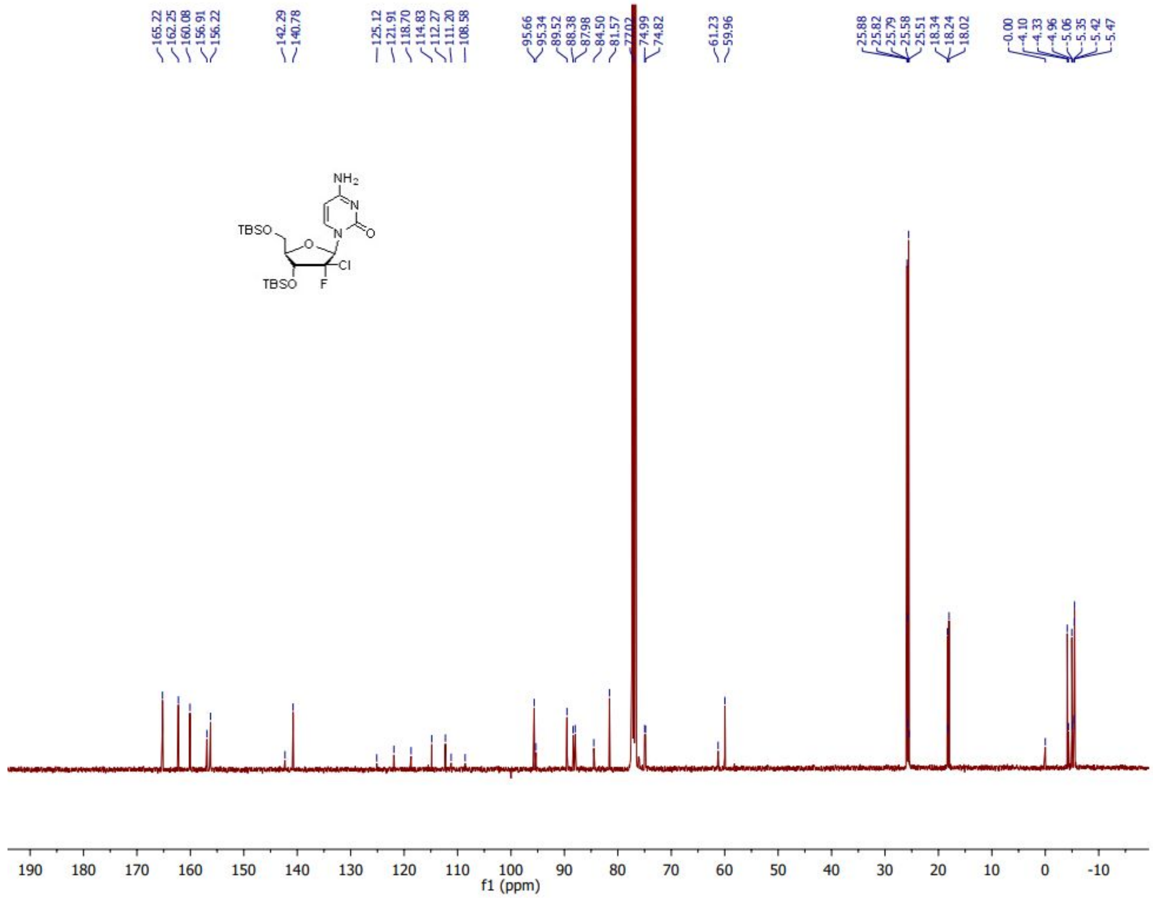
165.22
162.25
160.08
156.91
156.22
142.29
140.78
125.12
121.91
118.70
114.83
112.27
111.20
108.58
95.66
95.34
89.52
88.38
87.98
84.50
81.57
77.02
74.99
74.82
61.23
59.96
25.88
25.82
25.79
25.58
25.51
18.34
18.24
18.02
0.00
-4.10
-4.33
-4.96
-5.06
-5.35
-5.42
-5.47
NH2
TBSO
TBSO
F
Cl
f1 (ppm)

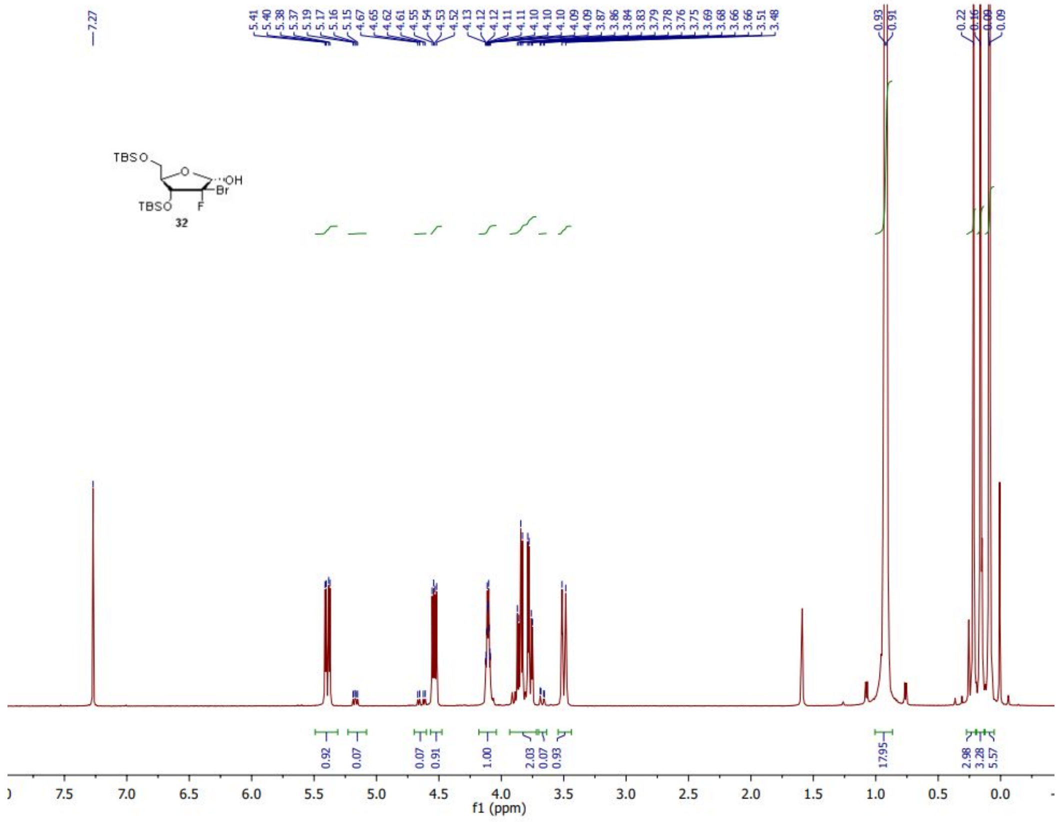
TBSO
OH
Br
TBSO
F
32
f1 (ppm)

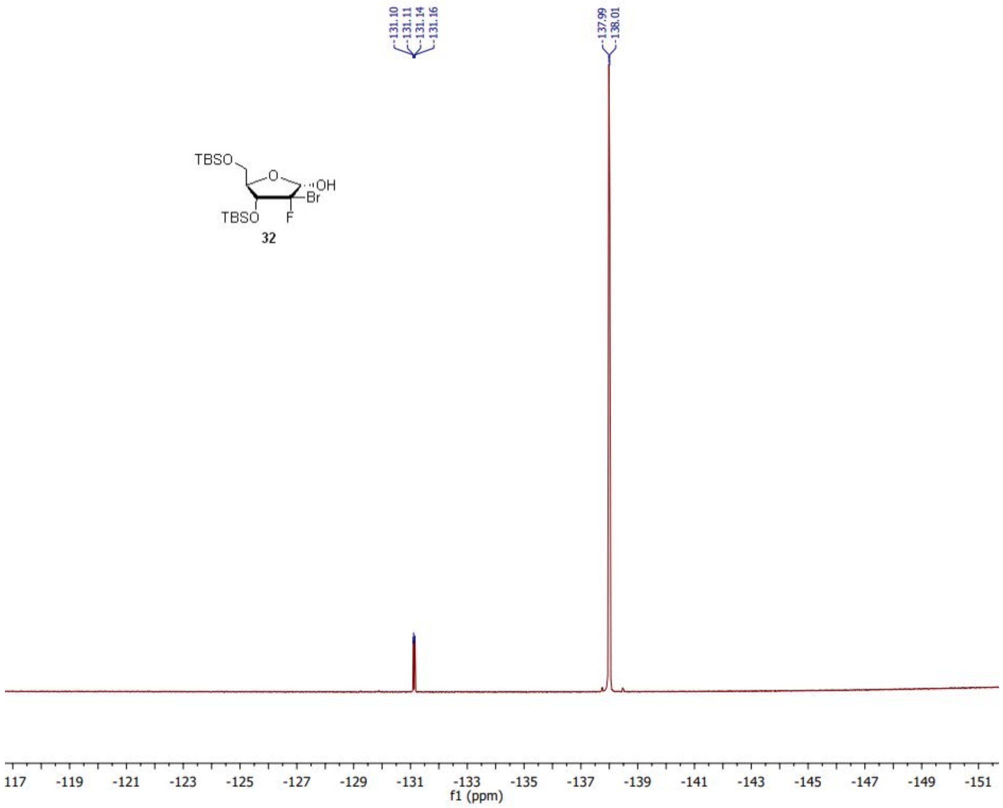
TBSO
OH
Br
TBSO
F
32
f1 (ppm)

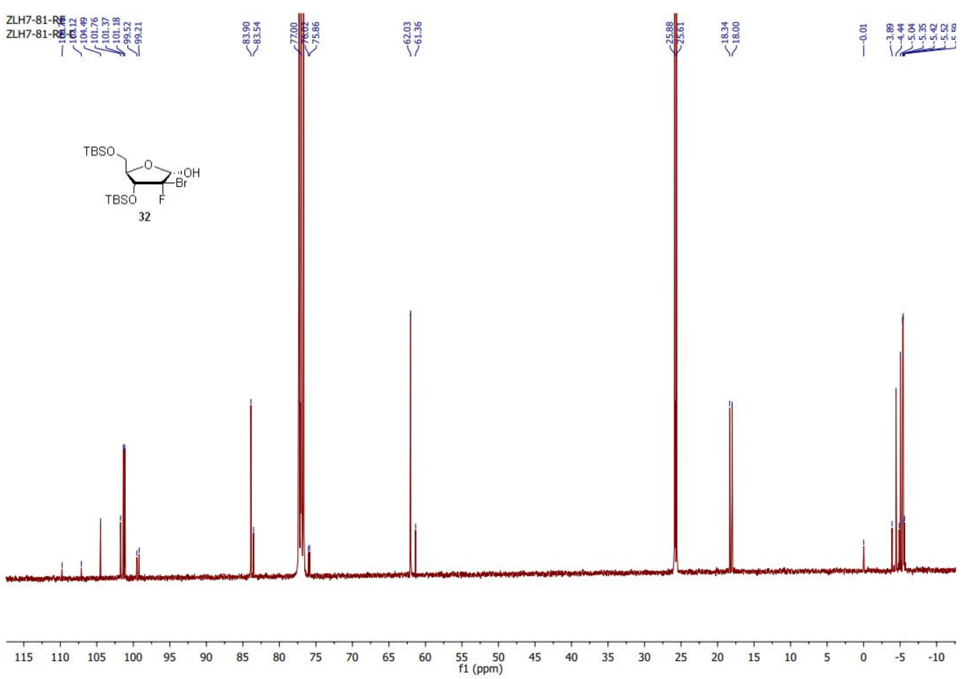
TBSO
OH
Br
TBSO
F
32
f1 (ppm)

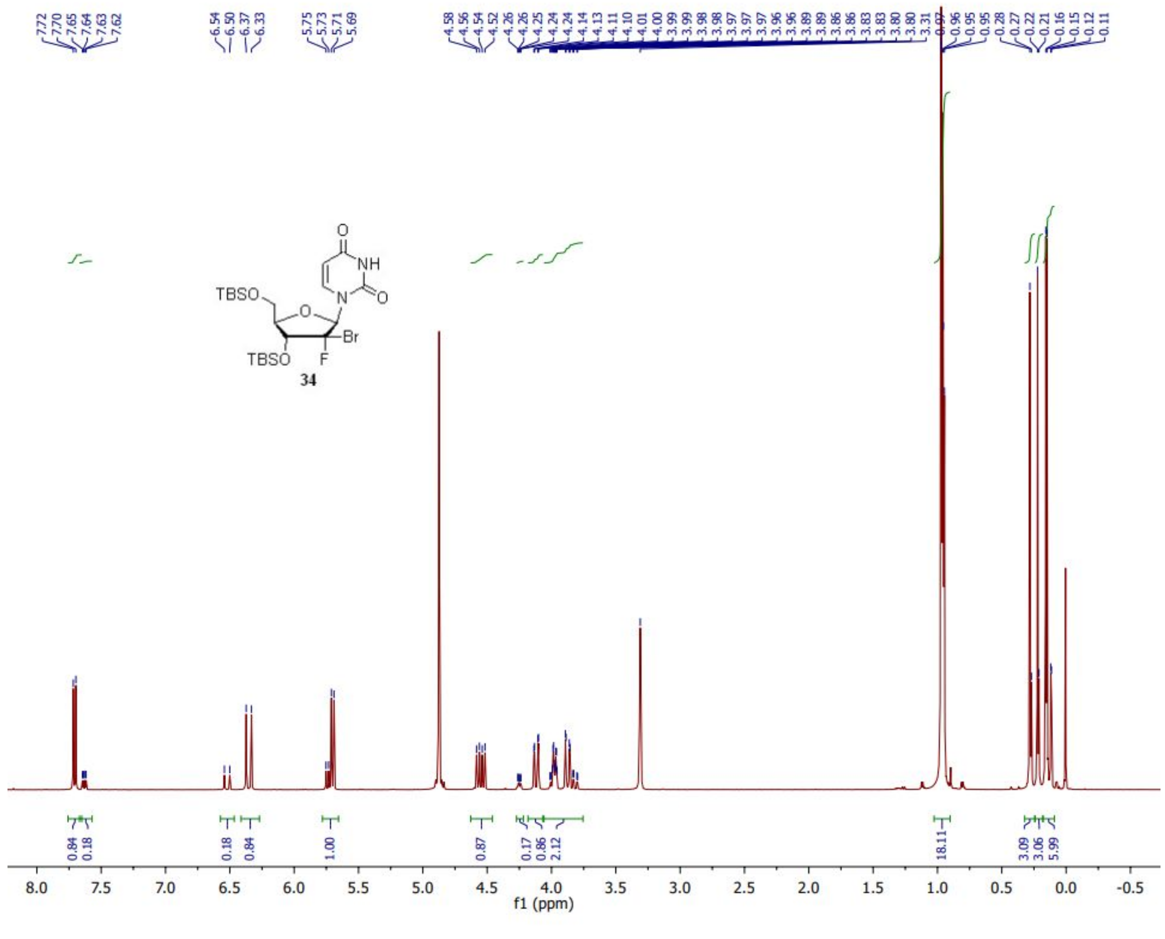
O
NH
N
O
TBSO
Br
TBSO
F
34
f1 (ppm)

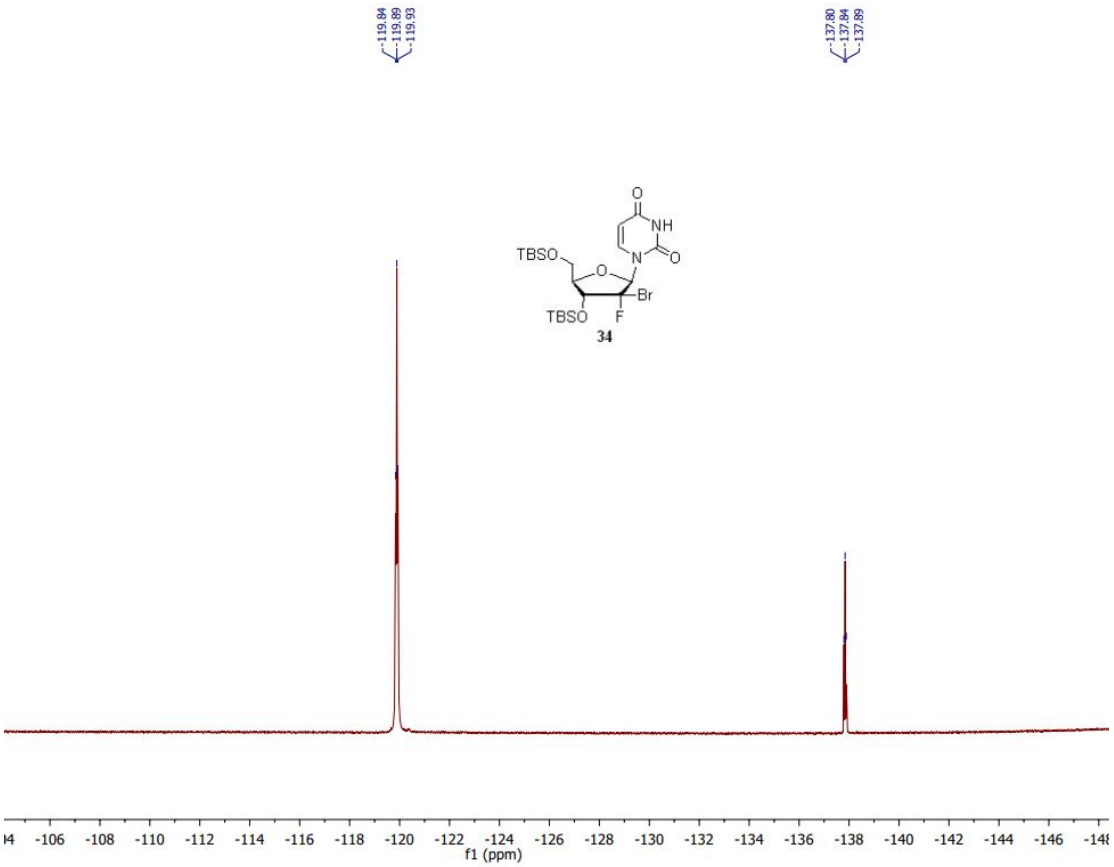

-119.84
-119.89
-119.93
-137.80
-137.84
-137.89
TBSO
NH
O
Br
F
TBSO
34
f1 (ppm)

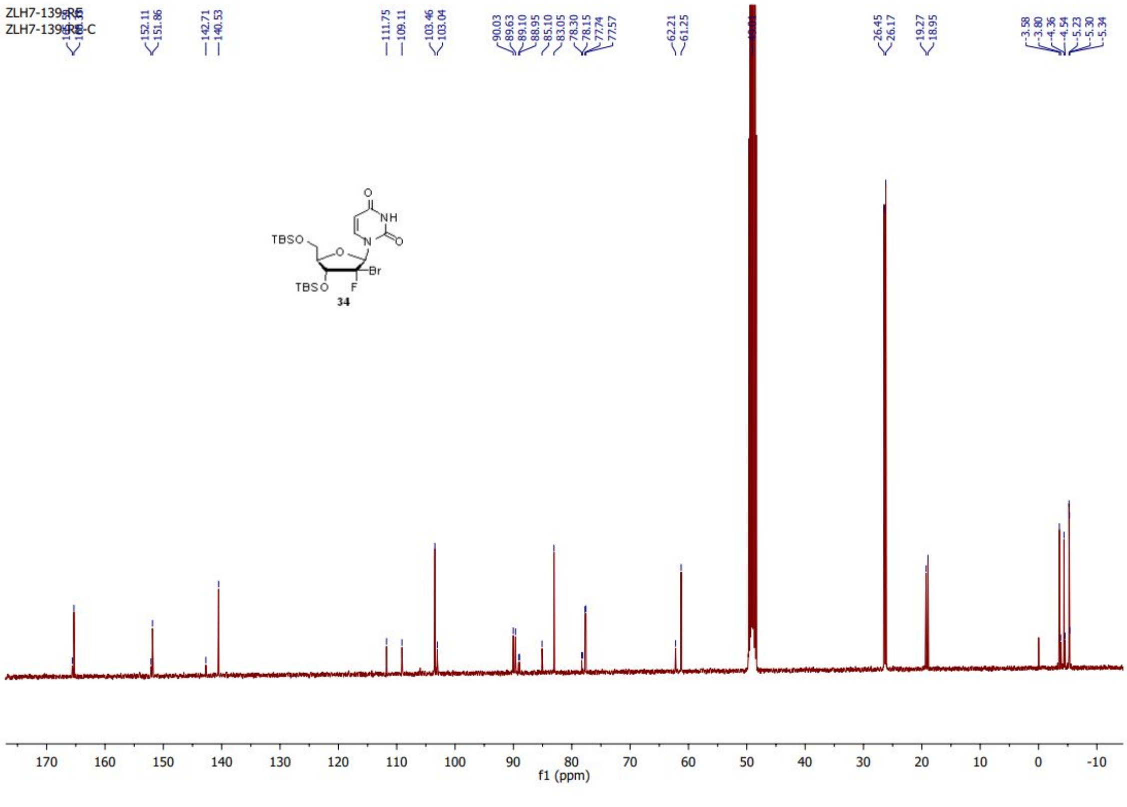

152.11
151.86
142.71
140.53
111.75
109.11
103.46
103.04
90.03
89.63
89.10
88.95
85.10
83.05
78.30
78.15
77.74
77.57
62.21
61.25
26.45
26.17
19.27
18.95
-3.58
-3.80
-4.36
-4.54
-5.23
-5.30
-5.34
TBSO
NH
O
Br
F
TBSO
34
f1 (ppm)

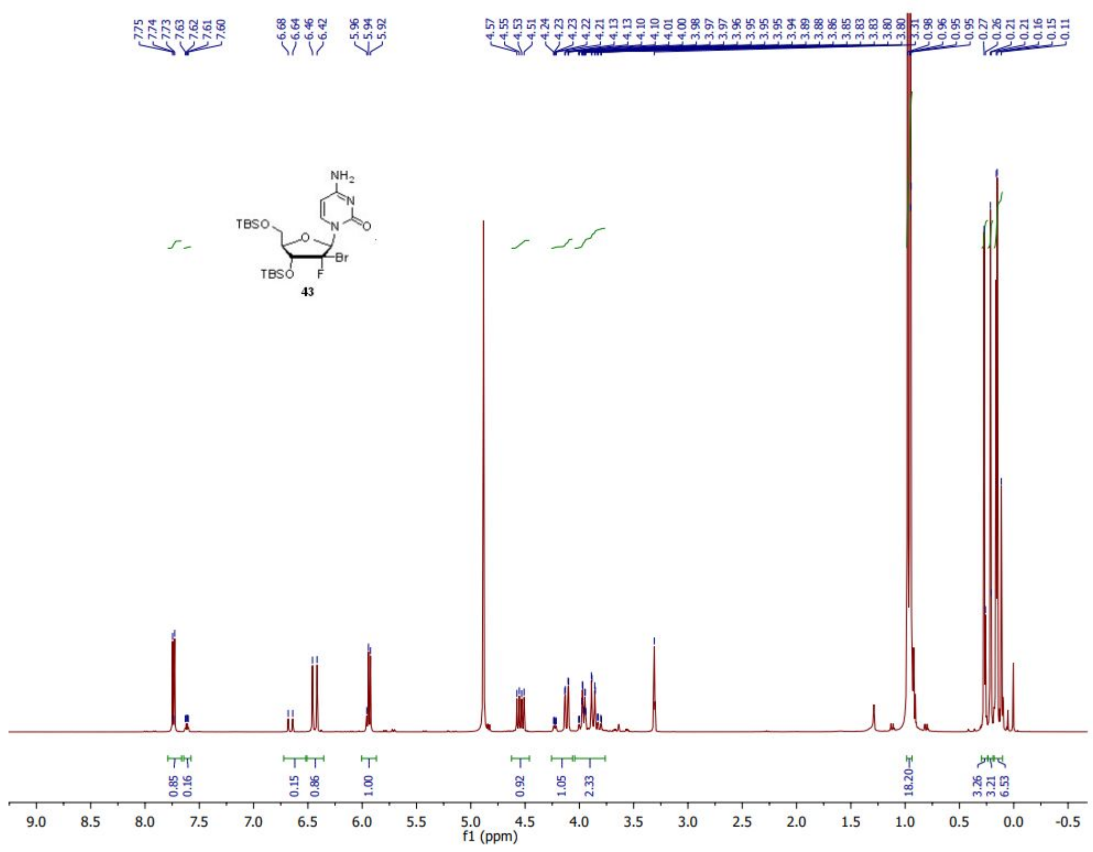
7.75
7.74
7.73
7.63
7.62
7.61
7.60
6.68
6.64
6.46
6.42
5.96
5.94
5.92
4.57
4.55
4.53
4.51
4.24
4.23
4.23
4.22
4.21
4.13
4.10
4.10
4.01
4.00
3.98
3.97
3.96
3.95
3.95
3.95
3.94
3.89
3.88
3.86
3.85
3.83
3.83
3.80
3.80
3.31
0.98
0.96
0.95
0.95
0.27
0.26
0.21
0.21
0.16
0.15
0.11
NH2
TBSO
TBSO
Br
F
O
N
43
0.85
0.16
0.15
0.86
1.00
0.92
1.05
2.33
18.20
3.26
3.21
6.53
9.0 8.5 8.0 7.5 7.0 6.5 6.0 5.5 5.0 4.5 4.0 3.5 3.0 2.5 2.0 1.5 1.0 0.5 0.0 -0.5
f1 (ppm)

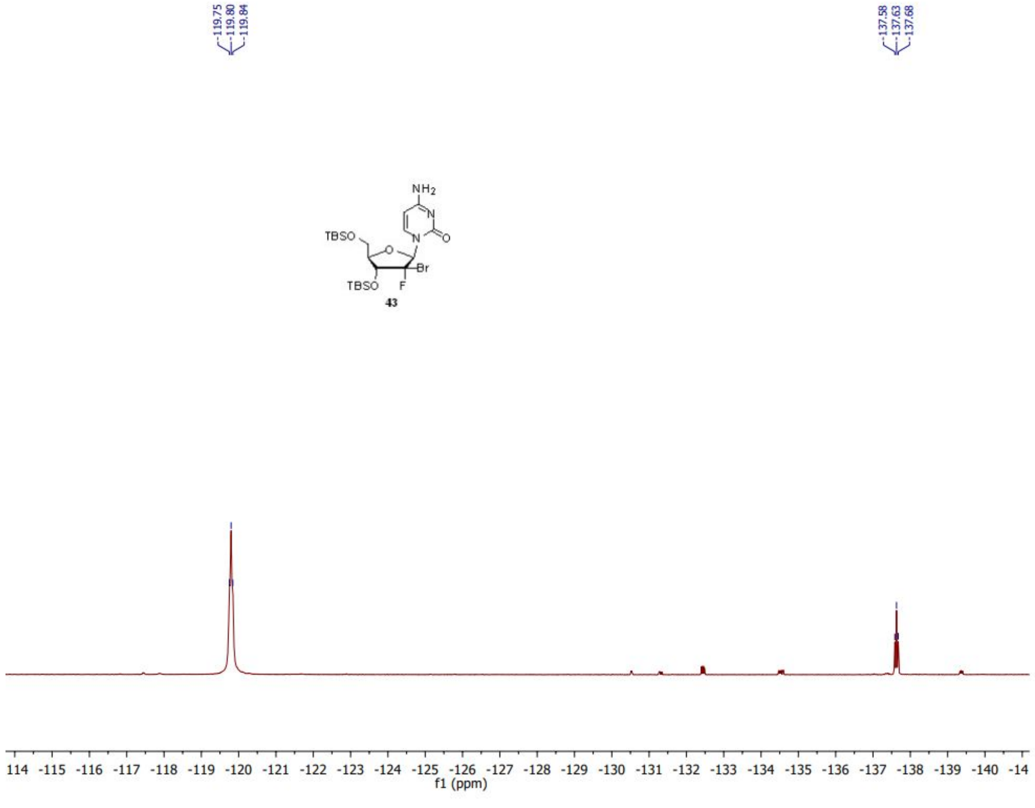
-119.75
-119.80
-119.84
-137.58
-137.63
-137.68
NH2
TBSO
TBSO
Br
F
O
N
43
114 -115 -116 -117 -118 -119 -120 -121 -122 -123 -124 -125 -126 -127 -128 -129 -130 -131 -132 -133 -134 -135 -136 -137 -138 -139 -140 -14
f1 (ppm)

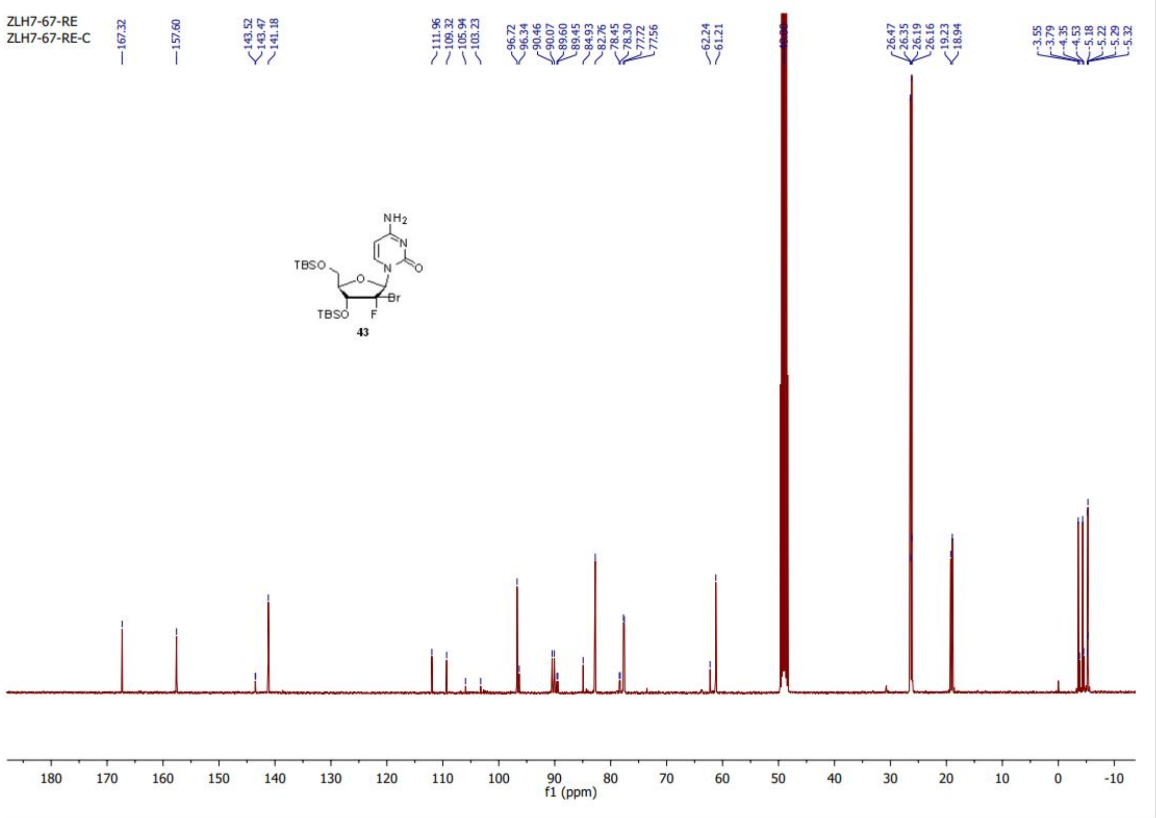
ZLH7-67-RE
ZLH7-67-RE-C
TBSO
TBSO
F
Br
NH2
43
f1 (ppm)

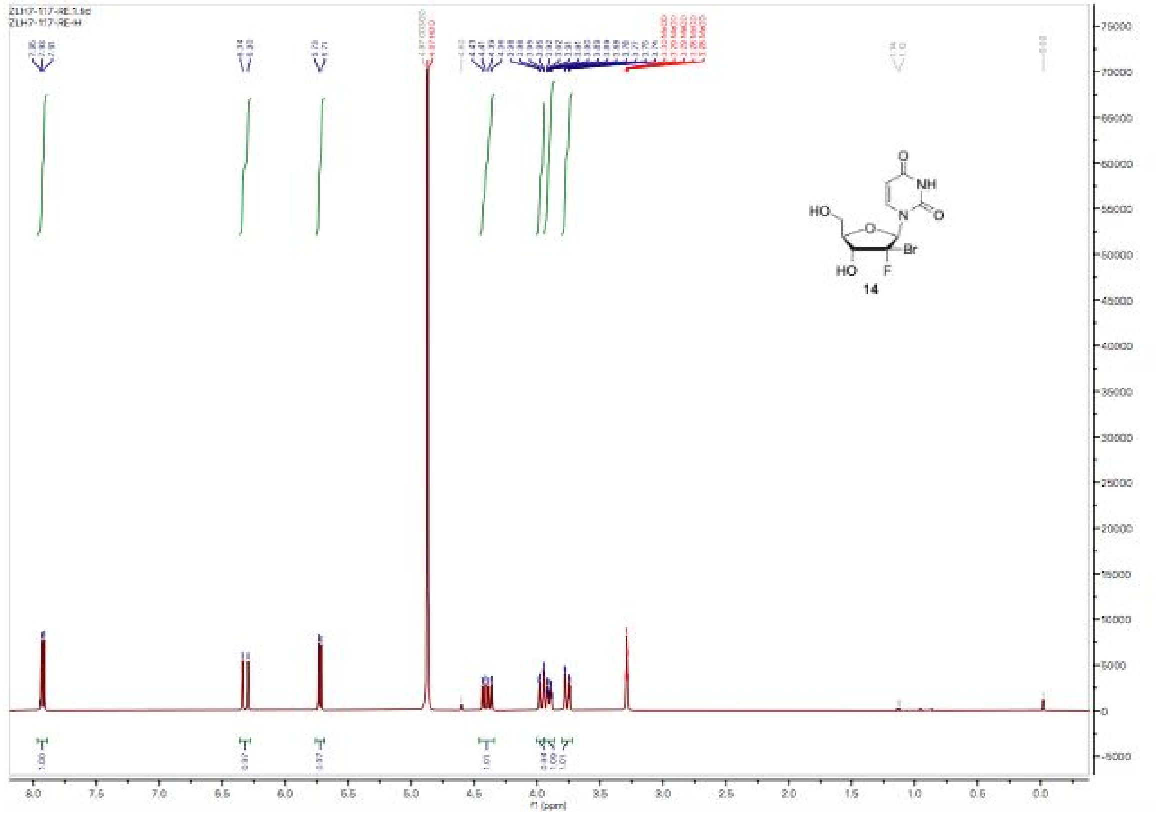
HO
HO
F
Br
NH
14
f1 (ppm)
